# Biomimetic hydrogel supports initiation and growth of patient-derived breast tumor organoids

**DOI:** 10.1038/s41467-022-28788-6

**Published:** 2022-03-18

**Authors:** Elisabeth Prince, Jennifer Cruickshank, Wail Ba-Alawi, Kelsey Hodgson, Jillian Haight, Chantal Tobin, Andrew Wakeman, Alona Avoulov, Valentina Topolskaia, Mitchell J. Elliott, Alison P. McGuigan, Hal K. Berman, Benjamin Haibe-Kains, David W. Cescon, Eugenia Kumacheva

**Affiliations:** 1 Department of Chemistry, University of Toronto, 80 Saint George Street, Toronto, ON M5S 3H6 Canada; 2 Princess Margaret Cancer Centre, University Health Network, 610 University Ave., Toronto, ON M5G 2M9 Canada; 3 Department of Medical Biophysics, University of Toronto, 101 College Street, Toronto, ON M5G 1L7 Canada; 4 Institute of Biomedical Engineering, University of Toronto, 4 Taddle Creek Road, Toronto, ON M5S 3G9 Canada; 5 Department of Chemical Engineering and Applied Chemistry, University of Toronto, 200 College Street, Toronto, ON M5S 3E5 Canada; 6 Department of Laboratory Medicine and Pathobiology, University of Toronto, 1 King’s College Circle, Toronto, ON M5S 1A8 Canada; 7 Department of Medicine, University of Toronto, Toronto, ON M5S 1A8 Canada

**Keywords:** Breast cancer, Biomaterials - cells, Gels and hydrogels, Breast cancer

## Abstract

Patient-derived tumor organoids (PDOs) are a highly promising preclinical model that recapitulates the histology, gene expression, and drug response of the donor patient tumor. Currently, PDO culture relies on basement-membrane extract (BME), which suffers from batch-to-batch variability, the presence of xenogeneic compounds and residual growth factors, and poor control of mechanical properties. Additionally, for the development of new organoid lines from patient-derived xenografts, contamination of murine host cells poses a problem. We propose a nanofibrillar hydrogel (EKGel) for the initiation and growth of breast cancer PDOs. PDOs grown in EKGel have histopathologic features, gene expression, and drug response that are similar to those of their parental tumors and PDOs in BME. In addition, EKGel offers reduced batch-to-batch variability, a range of mechanical properties, and suppressed contamination from murine cells. These results show that EKGel is an improved alternative to BME matrices for the initiation, growth, and maintenance of breast cancer PDOs.

## Abstract (web summary)

Patient-derived tumour organoids are important preclinical models but suffer from variability from the use of basement-membrane extract and cell contamination. Here, the authors report on the development of mimetic nanofibrilar hydrogel which supports tumour organoid growth with reduced batch variability and cell contamination.

## Introduction

Breast cancer is the most common cancer diagnosed in women worldwide and is responsible for substantial morbidity and mortality^1,2^. Development of effective breast cancer treatments is hindered by the lack of efficient preclinical models that recapitulate the complexity and heterogeneity of breast tumors in vivo. Breast cancer has many histologic and molecular subtypes, and individual cancers have distinct genotypes, morphologies, and treatment sensitivities, which are shaped by prior treatments^3,4^. Furthermore, the breast tumor extracellular matrix (ECM) is highly heterogeneous^5^ and its dynamic structure and physical properties influence tumor progression and treatment response^6^. In spite of this diversity, over the past several decades, breast cancer research has relied primarily on two-dimensional culture of only a few dozen clonal cell lines that fail to fully capture breast cancer heterogeneity, limiting their use in predicting clinical outcomes^7^.

Currently, patient-derived xenografts (PDX), in which human tumor fragments are transplanted directly into immunocompromised mice, serve as the gold-standard in fundamental and translational breast cancer research, as they largely retain the morphology, genomic profile, and intratumoral heterogeneity of the parental tumor^8^. Furthermore, drug response in PDX models appears to correlate well with the clinical response of donor patients^9,10^. Yet, PDX models present ethical challenges, and are cost, time, and labor-intensive. Furthermore, new PDX models can take months to years to develop, and for hormone-receptor positive breast cancers, tumor engraftment is highly inefficient.

Patient-derived tumor organoids (PDOs), sub-millimeter three-dimensional multicellular structures grown from cancer patient tissue in a three-dimensional matrix, have emerged as a promising model that bridges the gap between immortalized cell lines and PDX models^11^. In contrast to cell lines, the PDO models capture intra- and interpatient tumor heterogeneity and are significantly less resource-intensive than PDX models^12,13^. Furthermore, PDOs have the capacity to maintain the histological features and gene expression, and, most importantly, drug response of the donor patient tumor^14–19^, thus making them reliable models for preclinical evaluation of anticancer agents and potentially, personalized cancer therapies. Over the past 6 years, methods for PDO model generation have been reported for diverse solid tumors, including colorectal^19^, lung^20^, pancreatic^14^, ovarian^18^, prostate^21^, breast^16^, stomach^22^, and other solid tumors^11^, thereby rapidly making PDOs indispensable in vitro models.

Currently, pre-clinical PDO applications are hindered by their heavy reliance on mouse tumor basement membrane extract (BME) (commercially available as Matrigel, Cultrex BME or Geltrex), a “gold standard” hydrogel for 3D cell culture^14–19^. BME is a gelatinous mixture of laminin, type IV collagen, entactin, proteoglycans, and growth factors, which is secreted by Engelbreth-Holm-Swarm mouse sarcoma cells^23–25^. The presence of xenogeneic compounds and residual growth factors, as well as batch-to-batch variability in BME composition and properties leads to compromised reliability of BME as a matrix for PDO growth^16,18,21,22^. Furthermore, BME is not conducive to modifications of its mechanical properties, which are important for the development of an understanding of the role of mechanical and structural cues provided by the tumor microenvironment in cancer progression^6,26–29^ and tumor response to drugs^30–32^. Since BME is a physical hydrogel, it has poor tolerance of flow-induced stresses, thus complicating BME’s use in microfluidic organoid-on-a-chip platforms. Importantly, for the development of new organoid lines from patient-derived xenografts (PDXOs), contamination of murine host cells, which overtakes the human organoids in culture, poses a problem. Thus, a strong need exists for alternative biomimetic chemically crosslinked hydrogels for breast PDO initiation and maintenance, in order to extend the potential applications of this model system.

Several biologically-derived hydrogels formed by proteins (e.g., collagen^33^ or fibrin^28,29^) and polysaccharides (e.g., hyaluronic acid^31,32^ or alginate^34^), as well as synthetic matrices^35–39^ have been developed as matrices for spheroid growth from cancer cell lines and organoid culture. These spheroids, however, often do not reflect the biology and the clinical spectrum of primary cancer cells and cannot be used for prediction of patient-specific responses to therapy^7,40^. Since these hydrogels generally do not emulate the composition, structure, and properties of the extracellular matrix (ECM) in vivo^36,37^, many patient-derived cancer cells that are aggressive in vivo, do not grow in synthetic matrices in vitro as they lack the appropriate microenvironment^25,36^. In particular, the vast majority of synthetic hydrogels fail to recapitulate the filamentous architecture of the breast tumor ECM, which has a significant impact on cell mechanotransduction, growth factor signaling, long-distance cell-to-cell communication, and migration^38,41–43^. For the small fraction of hydrogel matrices that have been successfully used for PDO propagation from patient-derived breast and colorectal tumor cells^39,44,45^, the ability to initiate new PDOs lines and maintain them over multiple passages, while preserving their phenotype remains largely unexplored. Recognizing that the ECM in the breast tumor environment has a filamentous structure^6,38,46,47^ and a Young’s modulus in the range from 1.2 to 3.7 kPa^48^, we aim to design a chemically crosslinked biomimetic hydrogel recapitulating these properties.

Here we report a nanofibrillar hydrogel with controllable stiffness, which was prepared by the reaction between chemically modified cellulose nanocrystals and gelatin. This hydrogel (henceforth referred to as EKGel) provides the ability to grow and passage organoids initiated directly from patient tissue (PDOs) and PDX-derived tumor organoids (PDXOs) for multiple breast cancer subtypes. Comprehensive testing of PDOs grown in EKGel shows that they exhibit proliferation, histopathologic features, gene expression, and drug responses that are similar to those of the original tumors and to PDOs formed in standard BME. In contrast with BME, the EKGel exhibits strongly reduced batch-to-batch variability in mechanical properties and stability under close-to-physiological flow conditions, making it amenable to microfluidic “organoid-on-a-chip” platforms^49,50^. For generation of new PDO lines from primary patient material, EKGel matches BME’s initiation rate. However, for development of new organoid lines from PDXs (PDXOs), EKGel exhibits a distinct advantage. Whereas organoid culture in BME has been limited by contamination of murine host cells which can initiate and rapidly overtake the human organoids in culture^20^, here we show that EKGel allows for the initiation of PDXOs by suppressing contamination from murine cells. In summary, our results show that EKGel can replace BME matrices in the culture of breast cancer PDOs, enabling novel applications of organoid models and unlocking large collections of existing and well-characterized PDX for the development of breast PDXOs.

## Results

**Fig. 1:**
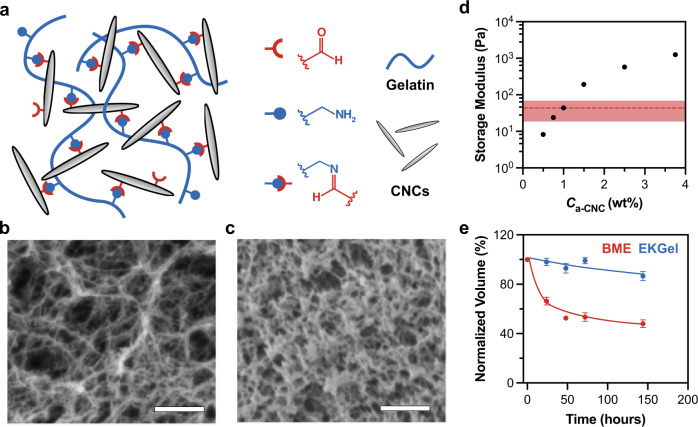
Properties of EKGel and BME. **a** Schematic of EKGel. **b**, **c** Scanning electron microscopy images of EKGel (**b**) and BME (**c**). Scale bars in (**b**, **c**) are 1 µm. In **b** *C*_*a-*CNC_ = 1 wt%. **d** Variation in the storage modulus, *G*’, of EKGel, plotted as a function of *C*_*a-*CNC_. Data shown as mean ± st. dev for *N* = 3 samples (error bars smaller than symbols). The shaded red area shows the standard deviation (*G*’ = 43 ± 24 Pa) for three distinct batches of BME. **e** Relative temporal volume loss of EKGel and BME under continuous perfusion of cell culture medium at a flow rate of 96 μm/s. In **e** data shown as mean ± st. dev of *N* = 50 microgels in a single experiment.

The biomimetic EKGel was synthesized from gelatin and rod-like aldehyde-modified cellulose nanocrystals (*a*-CNCs) with an average length and diameter of 176 ± 50 nm and 20 ± 4 nm, respectively. Figure 1a illustrates the hydrogel structure with Schiff base crosslinks between aldehyde groups on the *a*-CNC surface and amine groups of lysine residues in gelatin. Gelatin provided the arginine-glycine-aspartate integrin receptor-binding motif present in native ECM proteins, thus facilitating cell-matrix interactions^51^, while assembly of rod-like *a*-CNCs resulted in a nanofibrillar structure of the EKGel. EKGel is composed of a network of fibers (Fig. 1b), similar to the architecture of collagen in the in vivo tumor ECM^38,52–54^. The diameter of fibers in EKGel was from 20 to 105 nm with average fiber diameter of EKGel of 43 ± 17 nm (Supplementary Fig. 1), which was comparable with the dimensions of collagen fibrils in the breast tumor microenvironments^38,52–54^. The significantly larger pores in EKGel, in comparison with those in BME (Fig. 1c), enabled enhanced convection-driven transport of nutrients, waste products, and drugs through the EKGel matrix to tumor organoids^38,55^. The Darcy permeability of EKGel (a measure of convective transport) was 1.9 × 10^−11^ cm^2^, which was more than two orders of magnitude larger than the reported values for BME, varying from 10^−13^ to 10^−14^ cm^2^ ^56–58^.

The variation in mechanical properties of EKGel was achieved by changing *a*-CNC concentration, *C*_*a*-CNC_, in the hydrogel, while maintaining gelatin a concentration of 2 wt%. The storage modulus of EKGel was measured at 37 °C within the range of linear viscoelastic behavior (frequency of 1 Hz, 1 % strain, Supplementary Fig. 2). The shear storage modulus, *G*ʹ, of EKGel at 37 °C changed from 8 to 1246 with *C*_*a*-CNC_ increasing from 0.5 to 3.75 wt% (Fig. 1d), which corresponded to a Young’s modulus from 24 to 3738 Pa, respectively. This stiffness range covers the stiffness of ECM in breast tumor biopsies, which have Young’s modulus from 1.2 to 3.7 kPa)^59^. Importantly, for all compositions tested, the standard deviation for three distinct separately synthesized EKGel batches did not exceed 11% of the mean, while for BME (*G*ʹ = 43 Pa) for three separately purchased batches with different lot numbers the percent standard deviation was 56% (the shaded region in Fig. 1d). Notably, EKGel was not cytotoxic over the entire range of *C*_*a*-CNC_ in Fig. 1D (Supplementary Fig. 3). For the remainder of this work, including initiation and culture of breast PDOs we used EKGel with *Gʹ*  = 44 Pa to match the storage modulus of BME.

Covalent crosslinking of EKGel resulted in enhanced hydrogel stability, in contrast with BME. Figure 1e shows the decrease in EKGel and BME volumes under the continuous flow of cell culture medium at 96 μm/s. The geometry of the microfluidic device used to measure hydrogel stability is shown in Supplementary Fig. 4 and experimental details are provided in the Supplementary Information. Over five days, the relative reduction in BME and EKGel volume was 60 and 14%, respectively, which indicated higher EKGel stability under shear-induced stress, thus providing more consistent stiffness and porosity of the matrix over the relevant time period of PDO growth. EKGel experiences slow degradation as the imine crosslinks hydrolyze over time. In contrast, BME, which has no covalent crosslinking, is rapidly washed away. In the absence of flow, the reduction in volume for both matrices was minimal (Supplementary Fig. 4). This result indicates that EKGel is amenable for use in microfluidic organoid-on-a-chip platforms that incorporate physiological flow^50,60,61^.

To explore the versatility of EKGel for initiation and expansion of PDOs from primary breast cancer cells, we grew organoids from breast cancer cells with different receptor statuses and tissue sources, which were derived either from primary breast cancer tissue (PDOs) or from PDXs (PDXOs). As shown in Table 1, Lines 1 and 3 were developed from breast tumors obtained from patients that underwent surgical resection under informed consent, whereas Line 2 was PDX-derived. Breast cancer cells were isolated using a combination of mechanical disruption and enzymatic digestion (described in “Methods”).

**Table 1:** Characteristics of patient-derived breast cancer organoid lines.

Name	Abbreviation	Diagnosis	Tissue Source	Receptor Status	Initiation matrix
Line 1	PDO-1	Invasive ductal carcinoma	Primary breast cancer tissue	ER+(5–10%)/PR−/HER2−	BME
Line 2	PDXO-2	Metastatic breast cancer	Patient-derived xenograft	ER−/PR−/HER2−	BME
Line 3	PDO-3	Invasive ductal carcinoma	Primary breast cancer tissue	ER+(21–30%)/ PR+(21–30%) /HER2−	BME and EKGel

To initiate growth of PDOs, the isolated breast cancer cells were encapsulated in either EKGel, or BME by suspending cells in the hydrogel precursor suspension, and subsequently, allowing for gelation for 2 h. The cell-laden hydrogel was overlaid with breast cancer organoid media (Supplementary Table 1), and the cells were cultured for 2–3 weeks. To explore the ability of EKGel for PDO maintenance, PDO-1 and PDXO-2, which had been initiated and passaged 4 times in BME, were transferred into EKGel for subsequent passaging. PDO-3 was independently initiated in both BME and EKGel in parallel.

**Fig. 2:**
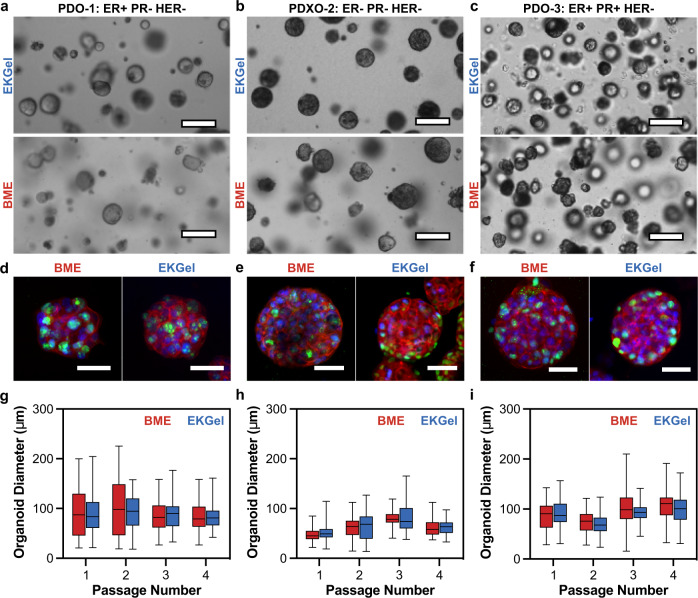
Growth of breast PDOs in EKGel and BME. **a**–**c** Brightfield images of PDOs grown in EKGel and BME from PDO-1 (ER+/PR−/HER2−) (**a**), PDXO-2 (ER−/PR−/HER2−) (**b**), and PDO-3 (ER+/PR+/HER2−) (**c**), as in Table 1. Scale bars are 100 µm. **d**–**f** Organoids in (**a**–**c**) stained for Ki67 (green), human EpCAM (red), and nuclei (blue). Scale bars are 50 µm. **g**–**i** Diameters of organoids formed in EKGel (blue) and BME (red) after four passages from PDO-1 (ER+/PR−/HER2−) (**g**), PDXO-2 (ER−/PR−/HER2−) (**h**), and PDO-3 (ER+/PR+/HER2−) (**i**). In (**g**–**i**) data shown as mean ± st. dev, with whiskers representing minimum and maximum values, of *N* = 100 spheroids measured over four repeated experiments. No significant difference between BME and EKGel observed (Student’s *t*-test, Bonferroni-Dunn method, two tailed, *p* > 0.01).

Figure 2a–c shows brightfield images of the PDOs grown in EKGel and BME from the Lines listed in Table 1. The PDOs in EKGel and BME grew into organoids consisting of spherical clusters of cells. Qualitatively, PDOs grown in BME and EKGel from each of three Lines had similar appearance. Figure 2d–f show the corresponding PDOs stained with antibodies directed against Ki67 and human EpCAM. The PDOs formed in BME and EKGel expressed human EpCAM on the cell surface, confirming that the cells are indeed human epithelial cells^62^. Similarly, the cell nuclei were positive for Ki67 staining, indicating that actively dividing cells are present after >2 weeks of culture^63^. Furthermore, no qualitative difference was observed in the number of focal adhesions or cytoskeleton organization between BME and EKGel (Supplementary Fig. 5).

To characterize PDO growth in EKGel and BME quantitatively, we monitored temporal change in organoid diameter and cell proliferation. Figure 2g–i show the diameters of the organoids formed from the three lines in BME and EKGel after each of four passages. No statistically significant difference was observed between the diameters of PDOs grown in EKGel and BME (Student’s *t*-test, Bonferroni-Dunn method, *p* > 0.01). Furthermore, the organoid diameters for each line were consistent over four passages, indicating that EKGel is suitable for long-term passage and maintenance of breast PDOs. Cell proliferation in BME and EKGel was monitored by counting the number of cells at each passage for four consecutive passages. The cell population doubling time for each of the three PDO lines ranged from 77 to 160 h, with no statistically significant difference for PDOs formed in BME and EKGel (Student’s *t*-test, Bonferroni-Dunn method, *p* > 0.01) (Supplementary Fig. 6).

To verify that organoid growth in EKGel does not influence the tumor initiating capability of the breast cancer cells, we initiated xenografts in immunocompromised mice from PDOs maintained in each matrix (Supplementary Fig. 7a). Tumors derived from organoids grown in EKGel and BME grew at similar rates, and both grew to a final volume of 1.3 cm^3^ by 144 days post-injection (Supplementary Figure 7b, *N* = 1).

**Fig. 3:**
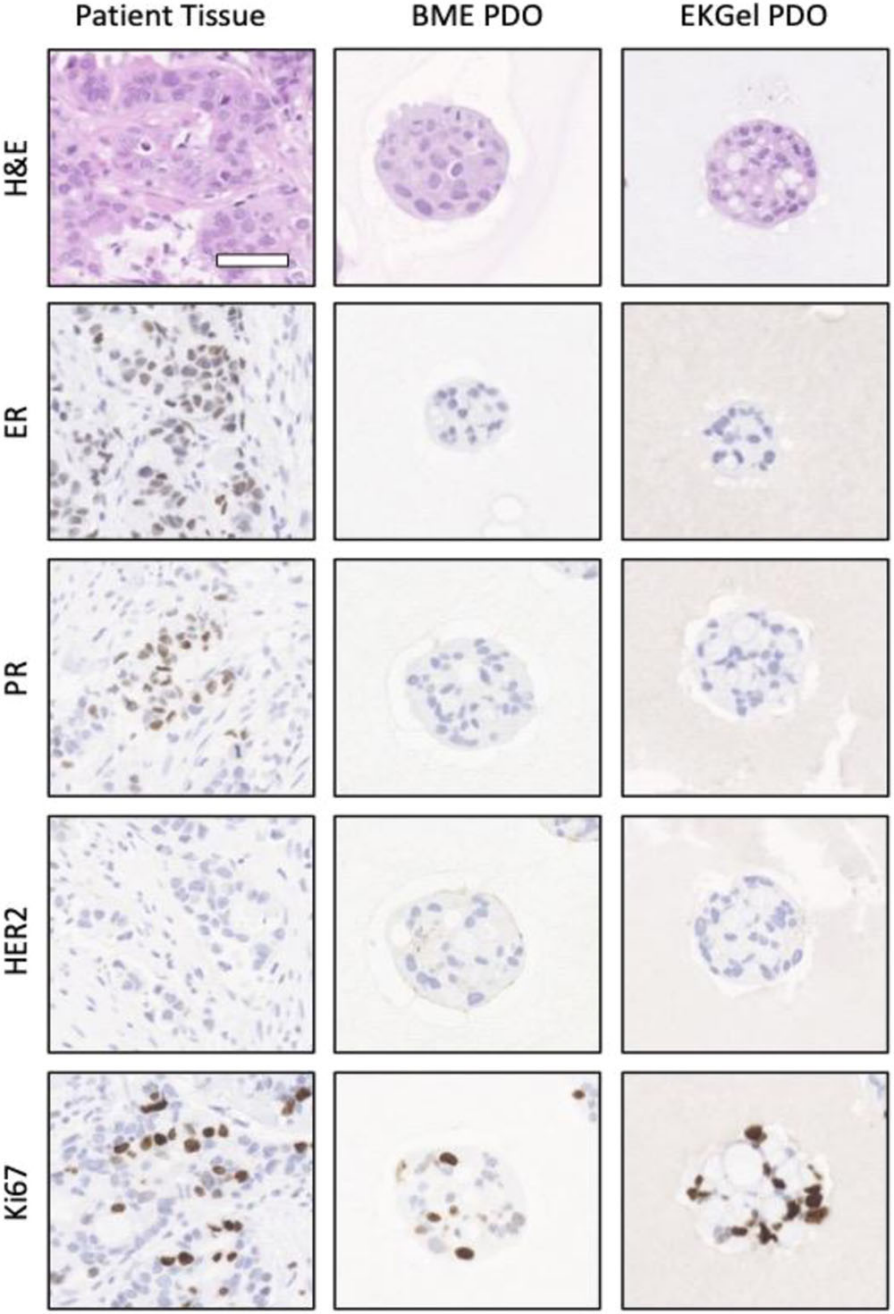
Tumor and organoid histology. Hematoxylin and eosin staining, and immunohistochemistry were performed on PDO-3 that were independently initiated and then passaged 4 times in BME and EKGel. Staining was performed in parallel with the primary tumor from which the organoids were derived. Scale bar is 100 µm.

Under optimal conditions, PDOs should recapitulate histologic features of their parental tumors and maintain protein expression of clinically relevant biomarkers. To further explore the suitability of EKGel for PDO formation, histology and biomarker immunostaining of organoids, grown in EKGel and BME, were analyzed and compared to their parental tumors by an experienced clinical breast pathologist (Analysis of PDO-3 in Fig. 3 and PDO-1 and PDXO-2 in Supplementary Fig. 8). In histologic sections for all samples studied, PDOs grown in EKGel and BME appeared equally well-formed and showed highly similar architecture and cytomorphology. Characteristic histologic features of the parental tumor were observed in both types of PDOs, including relative abundance of eosinophilic to amphophilic cytoplasm, varying degree of cytoplasmic vacuolization with focal formation of “clear-cells”, round to oval nuclei with moderate to high nuclear pleomorphism, dispersed chromatin pattern with focal vacuolation and variable formation of one to multiple prominent nucleoli (Fig. 3). As has been previously reported in organoid systems, for PDO-3 both EKGel and BME cultures exhibited reduced ER expression compared to the parental tumor, and was present in only a minority of organoids^16,64,65^. Similarly, PR expression (which was weak in the clinical specimen) was reduced in the organoids (Fig. 3). We did, however, observe heterogeneous ER expression in many of the PDO-1 organoids as seen in the patient tissue (Supplementary Fig. 8a).

**Fig. 4:**
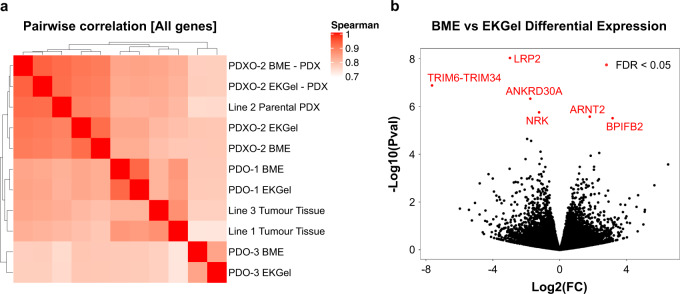
Gene expression of breast cancer organoids in EKGel and BME. **a** Pairwise correlation of global gene expression in PDOs grown in EKGel and BME, as well as associated in vivo xenograft or tumor tissues. For each Line, gene expression in BME and EKGel organoids grown in vitro or in vivo are highly correlated (average Spearman Correlation = 0.89). **b** Differential gene expression analysis to compare global gene expression for PDOs grown in vitro in BME vs. EKGel. Only six genes, as shown with red color, showed differential expression at false discovery rate (FDR) threshold of 0.05, none of which are recognized as important determinants of drug response or breast cancer biology.

To evaluate the similarity in gene expression between PDOs grown in different matrices, we performed RNA sequencing of cells from each condition (see “Methods”). RNA was extracted from cells isolated from organoid cultures or from frozen tumor tissue, libraries prepared after ribosomal RNA depletion and subjected to paired end sequencing to obtain approximately 80 million reads per sample on an Illumina Novaseq 6000. We then performed systematic analyses to identify any differences imposed by the growth matrix and contrast these to the originating tumors. PDOs originating from the same tumor grown in different matrices clustered together with high similarity (average Spearman Correlation = 0.89) showing minimal differences in expression patterns between the growth matrices (Fig. 4a). In addition, we observed high similarities between PDOs and their originating tumors (average Spearman Correlation = 0.83) supporting the concept that PDOs are good models to recapitulate patients’ tumors (Fig. 4a). Furthermore, we performed a differential expression analysis to evaluate the differences between PDOs grown in EKGel and BME matrices at the gene level. We found only six genes to be significantly different between the two growth matrices (Fig. 4b). Further inspection of these genes did not reveal any connections to important breast cancer biological processes or therapeutics. A query of a large and comprehensive pan-cancer pharmacogenomic database (PharmacoDB) identified no significant correlations between the expression of any of these six genes and drug response. Given the low number of differentially expressed genes between BME and EKGel and even lower number after multiple hypothesis correction, we applied pathway enrichment analysis based on a Hypergeometric test in order identify any biological processes that differ between BME and EKGel and we found no pathway exhibiting a significant difference (FDR < 0.05) out of 1604 biological pathways curated by REACTOME database^66,67^. Altogether results demonstrated consistency in gene expression between PDOs grown in EKGel and BME and identified no major alterations in the expression of relevant genes or pathways that would be expected to impact the use of models grown in EKGel for basic research or pharmacologic testing.

**Fig. 5:**
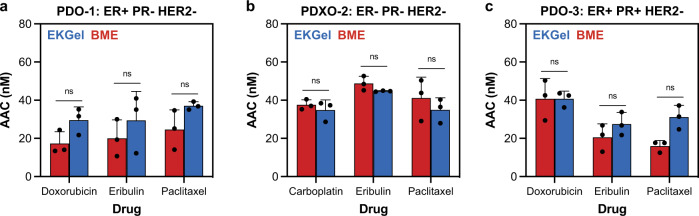
Response of breast tumor organoids to drugs. **a**–**c** Area above the curve (AAC) of three different drugs in EKGel and BME, measured by the cell titer-Glo assay. **a** AAC of eribulin, paclitaxel, and doxorubicin for PDO-1. **b** AAC of eribulin, paclitaxel, and carboplatin for PDXO-2. **c** AAC of eribulin, paclitaxel, and doxorubicin for PDO-3. In (**a**–**c**) data shown as mean ± st. dev of *N* = 3 biological replicates and ns indicates no significant difference (Student’s *t*-test, Bonferroni-Dunn method, two tailed, *p* > 0.01).

Next, we performed in vitro drug assays to explore the chemosensitivity of the organoids Lines listed in Table 1 grown in EKGel and BME to commonly used breast cancer drugs. Figure 5a–c show drug sensitivity measured as area above the curve (AAC) - a metric preferred for its reproducibility across pharmacogenomic studies^68,69^—for paclitaxel, eribulin, carboplatin, and doxorubicin in PDO-1, PDXO-2 and PDO-3, respectively. The dose–response curves used to determine AAC are included in Supplementary Fig. 9. No significant difference in the AAC values was observed between matrices (Student’s *t*-test, Bonferroni-Dunn method, *p* > 0.1).

To evaluate whether PDO growth in EKGel impacts their chemosensitivity in vivo, the organoids grown in EKGel and BME from PDXO-2 (ER-/PR-/HER2-) cells were enzymatically digested by TrypLE Express (Gibco) and the cells were implanted into the mammary fat pad of non-obese diabetic (NOD)/SCID mice to generate xenografts, which were subsequently treated with paclitaxel. Supplementary Fig. 10A shows a schematic illustrating this experiment. Once the tumors were established and reached a volume of ~150 mm^3^, paclitaxel treatment was initiated and delivered intravenously at an established and clinically-relevant dose (20 mg/kg), on a weekly schedule. The animals were sacrificed when the tumors reached the humane endpoint (1500 mm^3^). Supplementary Fig. 10B, C show the growth of tumors with and without drug administration in EKGel (EKGel-tumors) and BME (BME-tumors), respectively. The untreated controls grew at similar rates in both EKGel and BME. Both the EKGel-tumors and BME-tumors showed response to paclitaxel, with substantial tumor growth inhibition, but not regression observed in the drug-treated tumors. Furthermore, the growth rate of the paclitaxel-treated EKGel-tumors and BME-tumors were similar, indicating that the in vivo drug response appears unimpacted by organoid growth in EKGel vs. BME.

**Fig. 6:**
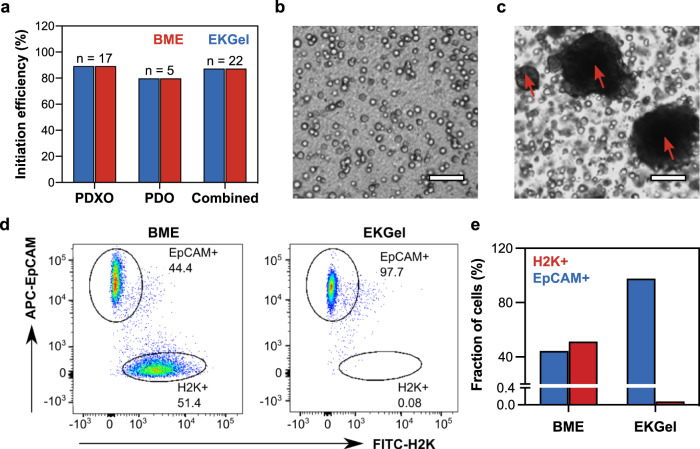
Organoid initiation in EKGel and BME. **a** Initiation rate of PDXO (*N* = 17) and PDO (*N* = 5) in EKGel and BME. **b**, **c** Brightfield microscopy images of PDXOs grown in EKGel (**b**) and BME (**c**) for 14 days. Scale bars are 100 μm. Red arrows indicate contaminating mouse cell clusters. **d** Flow cytometry characterization of the content of human (EpCAM+) and mouse cells (H2K+) of the PDXO shown in (**b**, **c**) initiated in EKGel (right) and BME (left). FITC-H2K was used to stain mouse cells and APC-EpCAM was used to stain human cells. **e** Comparison of the fraction of cells that are H2K+ or EpCAM+ for PDXO initiated in BME and EKGel in (**b**, **c**).

After verifying that organoids can be successfully initiated and grown in the EKGel, while maintaining the phenotype of their tumor of origin, we explored the potential of EKGel for initiation of new breast cancer PDOs and PDXOs. We processed multiple tumors from PDXs (*N* = 17) or directly from patients (*N* = 5), plated dissociated single tumor cells in parallel in EKGel and BME, and monitored organoid initiation. As shown in Fig. 6a, the overall initiation rate for organoids in both matrices was similar, that is, 88%  for PDXO and 80%  for PDO. Notably, one PDXO line (BPDXO.107) was successfully initiated in EKGel, but not in BME and another line, BPDXO.113, was initiated in BME, but not in EKGel, indicating that the initiation properties are not identical.

Although not extensively described in the literature, the establishment of PDXO models is confounded by the presence of murine host cells, which have the potential to grow and rapidly overtake the human organoids in culture. We and others have found such contaminations to be a major hurdle in the ability to establish long-term organoid cultures from PDX tumors (from breast and other cancers) and have therefore incorporated a mouse cell depletion step in an attempt to alleviate this problem^20,70,71^. Even with this purification step, we found a striking difference between organoid initiation from PDX tumors in BME and EKGel. Out of 17 of the PDX tumor organoids initiated in BME, 8 (or 47%) had significant mouse cell contamination, as determined by organoid imaging and/or by flow cytometry (Fig. 6). Mouse cell contamination appears as larger dark clusters that are indicated by red arrows in Fig. 6c.

Supplementary Fig. 11 shows immunofluorescence staining for Human EpCAM and mouse H2K-d that confirms that these clusters are indeed murine cells. In contrast, all of the same 17 PDX tumor organoids grown in EKGel were free of such mouse cell contamination. A representative example is shown in Fig. 6b–e. Dissociated, mouse cell depleted PDX tumor cells were plated in parallel in EKGel (Fig. 6b) and BME (Fig. 6c) and grown for 14 days. Flow cytometry was then performed on the cells to determine the mouse and human cell content. Figure 6d and e shows the fraction of EpCAM+ human cells and H2K+ mouse cells in from the same sample initiated in either EKGel and BME. The sample initiated in BME was 51.4% H2K+ mouse cells, while the sample plated in EKGel only contained 0.08% H2K+ mouse cells. Immunofluorescence staining for human EpCAM and mouse H2K-d confirmed that when PDX samples are plated in BME, both human organoids and large clusters of mouse cells are observed (Supplementary Fig. 11). In contrast, when the same PDX sample was plated in EKGel, only human EpCAM positive organoids, and no clusters of mouse H2K-d positive mouse cells were observed. Notably, single mouse cells were present when the PDX sample was plated in EKGel, but they did not proliferate into large mouse cell cultures that overtook the human organoids, as was observed in BME. We speculate that the increased proliferation of normal mouse cells in BME is related to the presence of residual murine growth factors, including TGF-β, IGF, PGFD, EGF, NGF, and VEGF in BME matrices^23,72,73^.

To further investigate the origins of EKGel’s ability to exclude contamination of mouse cells, we cultured different types of mouse cells in EKGel and BME. The PDXs used here are initially grafted into the mammary fat pad and then grafted subcutaneously for subsequent passages. To assess whether EKGel inhibits proliferation of normal (non-cancerous) mouse cells from skin or mammary tissue, we isolated cells from both a mammary gland and skin samples from mice and cultured them in both EKGel and BME (Supplementary Figs. 12 and 13). Proliferation of both mouse mammary gland and skin cells were significantly higher in BME than in EKGel. We speculate that the increased proliferation of normal mouse cells in BME is related to the presence of residual murine growth factors, including TGF-β, IGF, PGFD, EGF, NGF, and VEGF in BME matrices^23,72,73^. We propose that the difference in only the proliferation of the normal mouse cells, and not the human breast cancer cells, is that cancer cells have mechanisms to escape dependence on growth factors like IGF^72^, and thus do not require their presence for proliferation. To support this conclusion, we showed that there was no difference in the proliferation of mouse mammary tumor cells in BME and EKGel (Supplementary Fig. 14).

We next investigated if the mouse cell depletion step could be eliminated altogether when plating cells directly in EKGel. Five independent PDX tumors were processed for organoids and depleted and undepleted cells were plated in parallel in both BME and EKGel. Of the 5 PDX tumors, none of those that were undepleted of mouse cells had mouse cell contamination EKGel, while 4 out of 5 had contaminating murine cells when plated in BME (Supplementary Fig. 15). This observation reveals a significant advantage of EKGel over BME, as there are hundreds of breast cancer PDXs lines that have been established, characterized and made available through various consortia worldwide^74^. This important property of EKGel creates the opportunity to leverage the major investments made in these PDX resources to generate well-annotated organoid models.

## Discussion

We have developed a new biomimetic hydrogel for effective initiation and expansion of breast cancer PDOs and benchmarked its properties against BME, a “gold standard” hydrogel for 3D cell culture. EKGel had highly reproducible mechanical properties, with storage modulus fine-tuned over three orders of magnitude, thus overcoming the batch-to-batch variability and narrow range of stiffnesses of BME. While in most of our work we matched EKGel stiffness to that of BME, the mechanical properties of EKGel can be varied to replicate the properties of normal breast tissue and breast tumors^48,59^. Furthermore, EKGel has a fibrous structure that mimics the architecture of the tumor extracellular matrix^47^. This is a distinct advantage of EKGel, as fibrillar architecture of microenvironments have a significant impact on cell phenotype. Such architecture impacts mechanotransduction, growth factor signaling, long-distance cell-to-cell communication, and cancer cell invasion^41–43^. While several filamentous hydrogels have been developed, none have been validated for culture of breast PDOs to the extent we have shown here^38^. While the simplicity of EKGel preparation is its great advantage, further chemical functionalization of CNCs with *e.g*., proteoglycans can be readily achieved. Importantly, in comparison with BME (and other physically crosslinked gels), the EKGel was mechanically stable under close-to-physiological flow conditions, which enables its utilization in microfluidic organoid-on-a-chip models that are rapidly finding a broad range of applications in fundamental cancer research and drug screening^49,61,75–77^. This trend is driven by a recognition of the influential role that interstitial fluid flow plays in determining cell phenotype, tumor progression, and drug response^78–81^. The compatibility of EKGel with these platforms is a significant benefit, relative to BME.

We showed that EKGel supports both initiation and passage of breast cancer PDOs with different histologic subtypes and from different source materials (that is, both patient samples and PDXs), thereby demonstrating the versatility of EKGel as a matrix. Importantly, the growth, tumorigenicity, and drug response of the breast PDOs were consistent between EKGel and BME. Furthermore, no major alterations of the histopathological properties or gene expression patterns were identified between the source tissues and the PDOs grown in EKGel and BME. We did, however, observe a loss of ER and PR expression in the PDO-3 organoids grown in both matrices as compared to the tumor tissue from which they were derived. Loss or reduction in hormone receptor expression in breast cancer organoids relative to parental tissue has been described by others^16,64,65^. This phenomenon may be due to a combination of factors, including but not limited to clonal selection and media composition. The commonly used media formulation is serum-free and therefore, lacks estrogen, potentially explaining the loss/reduction in ER and PR, whose expression are hormonally regulated. Together, these results support the use of breast cancer organoids cultured in EKGel as an in vitro model for translational research, with potential for applications in personalized cancer therapy. In the future, we hope to validate the relevance of the in vitro drug response of PDOs to the in vivo and clinical settings. Importantly, such verification would be possible owing to EKGel’s ability to initiate PDXOs, as drug response can be compared to data from the source PDX. While the growth, tumorigenicity, and drug response of the breast PDOs were consistent between EKGel and BME, these results are limited by their n-of-1 design, and larger-scale studies will be required to fully characterize the relationship between in vitro and in vivo behaviors. Here we demonstrated that PDO properties are maintained in both EKGel and BME for up to four passages. In future work, it will be valuable to investigate the stability of PDO properties over long-term passage. We note that two models including PDO-3 have been initiated and passaged independently in BME and EKGel for over 10 passages.

Low initiation rates remain a problem with cancer PDOs and developing new matrices that can increase initiation rates would be a substantial advance^13^. Here, we show that initiation rate of new breast tumor organoid lines is the same for both EKGel and BME. The combined initiation rate for both PDOs and PDXOs in this work is 86% (*N* = 22), which is slightly higher than the previously reported initiation rate for breast cancer PDOs (exclusively from clinical samples) of ~80%^16^. Notably, the ability to tune the physical properties of EKGel could provide a route to improve breast cancer organoid initiation rates in the future. Future work should explore initiation rates in hydrogels with different stiffnesses. Furthermore, the ability to vary and control the stiffness of EKGel over a broad range opens the door for studies on the effect of the mechanical properties of the matrix on PDO properties.

Importantly, the EKGel used here exhibited a distinct advantage for the initiation of PDXOs by suppressing the contamination and overgrowth of murine cells that is commonly observed in BME. This advantage unlocks the hundreds of well-characterized PDXs around the world as tissue sources for organoid development, and in vitro applications that are not feasible to carry out in vivo. In addition, the absence of xenogenic components in the culture system may facilitate the application of PDO to study interactions between cancer and immune cells, a critical need for the ongoing development of immuno-oncology therapies.

The ability of EKGel to suppress the overgrowth of normal mouse cells deserves special attention, as it suggests that EKGel may also suppress overgrowth and contamination by normal human cells. This effect may offer a crucial advance: when initiating PDOs, normal human cells present in the source tumor tissue source often overtake cancer cells in PDO cultures, thus posing a challenge in the application of PDOs in personalized medicine^13,82^. If EKGel can suppress the overgrowth by normal human cells, it would provide a route to overcoming this limitation of PDOs. This interesting and potentially, very useful application of EKGel—under appropriate conditions—will form the basis of a separate study.

Organoids have been established from a broad range of tissue types, including both healthy tissue, primary tumors, and metastatic lesions. Based on our results, EKGel has the potential to serve as a scaffold for these organoid types, enabling in vitro models that capture intra- and interpatient heterogeneity that could be used to develop personalized cancer therapies. Future large-scale evaluation of this novel biomaterial will permit more detailed characterization of the degree to which EKGel permits faithful organoid modeling across diverse breast and other cancer types and permits accurate prediction of clinical response to diverse drug classes.

## Methods

### Materials

Type A gelatin (300 g bloom), ethylene glycol (≥99% purity), 25% glutaraldehyde aqueous solution (≥98% purity), and sodium periodate (≥99 % purity) were purchased from Sigma-Aldrich, Canada. An aqueous 12.2 wt % CNC suspension was purchased from the University of Maine Process Development. BME was purchased from Trevigen, Inc. All chemicals were used as received without further purification unless otherwise specified. All other reagents (drugs, assay reagents, etc.) are listed in Supplementary Table 3, and antibodies are listed in Supplementary Table 4. Breast tumor material was obtained following informed consent and used under Research Ethics Board-approved protocols at the Princess Margaret Cancer Centre (UHN #15-9481).

### Preparation of breast organoid medium

A detailed list of the components is provided in Supplementary Table 1. Organoid media was stored at 4 °C. Breast organoid medium composition was prepared as described elsewhere^16^.

### Surface modification of CNC with aldehyde groups

Aldehyde-functionalized CNCs (*a*-CNCs) were prepared by surface oxidation of CNCs with sodium periodate, as reported elsewhere (*54*, *68*). Sodium periodate (NaIO_4_, 1.2 g) was added to 200 mL of a 1 wt % CNC suspension. The flask was covered with aluminum foil, and the mixture was stirred for 2 h at room temperature. The oxidation reaction was quenched by adding 600 µL of ethylene glycol. The suspension of *a*-CNCs was dialyzed for 2 weeks against Milli-Q grade deionized water (DI, 18.2 MΩ cm resistivity) with the water being changed twice a day (cellulose membrane, 12 kDa cutoff). The *a*-CNC suspension was then concentrated to >3 wt% using rotary evaporation. To adjust the ionic strength of *a*-CNC solution, 10× HBSS buffer was added to the *a*-CNC suspension in a 1:10 vol. ratio to reach a final concentration of 1× HBSS.

### Preparation of EKGel

EKGel was prepared by thorough vortex mixing of a stock *a*-CNC suspension (3 wt% in HBSS) and gelatin solution (10 wt% gelatin in Advanced DMEM/F12 cell culture medium) with organoid medium to reach a final composition of 1 wt% *a*-CNC and 2 wt% gelatin. The stock suspensions were sterilized under ultraviolet light for at least 10 min and kept in a 37 °C water bath for at least 20 min prior to gelation, to ensure that gelatin did not undergo physical gelation at low temperatures.

### Scanning electron microscopy

The structures of EKGel and BME were studied using scanning electron microscopy (SEM). Supercritical point drying was utilized to prepare hydrogel samples. EKGel samples were allowed to gel overnight at 37 °C, fixed by submerging them in 2 wt % glutaraldehyde in HBSS for 24 h and washed with deionized water three times. Subsequently, the water was exchanged with ethanol by consecutively submerging the EKGel for 30 min in 30, 40, 50, 60, 70, 80, and 90 % (v/v) ethanol/water mixtures and then finally, in pure ethanol. Afterward, the hydrogels were placed in an Autosamdri-810 Tousimis critical point dryer. The ethanol in the sample was exchanged with liquid CO_2_, which was subsequently brought to a supercritical state and removed by slow venting. The dried EKGel was fractured and gold-coated using an SC7640 High Resolution Sputter Coater (Quorum Technologies). The samples were imaged on a Quanta FEI scanning electron microscope (10 kV).

### Rheology

The rheological properties of the EKGel and BME were characterized using a rheometer (AR-1000 TA Instruments) with a cone and plate geometry, with a cone angle and diameter of 0.9675° and 40 mm, respectively. An integrated Peltier plate was used to control the temperature, and a solvent trap was utilized to minimize solvent evaporation. The hydrogels were allowed to equilibrate at 37 °C for 3 h before experiments. Unless specified, a strain was 1% and frequency of 1Hz were used (within the linear viscoelastic regime) and the temperature during the measurements was 37 °C.

### Determining EKGel permeability

The Darcy permeability coefficient, *K*_s_, of EKGel was determined using a previously reported method^57,58^. Briefly, an EKGel sample with dimensions 3 mm × 3mm × 13.7 mm (width × height × length) was prepared in a chamber fabricated in poly(dimethylsiloxane) (PDMS). The chamber was connected to inlet and outlet reservoirs by perfluoroalkoxyalkane tubing (IDEX Health & Science). The inlet reservoir was placed above the outlet reservoir to apply a difference in pressure, Δ*P*, to the hydrogel. The HBSS solution was perfused through the hydrogel under Δ*P*. The volumetric flow rate, *Q*_p_, of this solution was determined by measuring the change in the mass of the outlet reservoir over time. The Darcy permeability coefficient, *K*_s_, was determined by Eq. 1, where *L* is the hydrogel length (13.7 mm); Δ*P* is the pressure difference across the hydrogel, calculated from the difference in heights of the inlet and outlet reservoirs; *η* is the viscosity of HBSS solution (taken as 1.002 cP), and *A* is the cross-sectional area of the hydrogel (9 mm^2^).

1 $${K}_{{{{{{\rm{S}}}}}}}=\frac{\eta L{Q}_{p}}{A\Delta P}$$

### Patient tumor dissociation

Patient tumor tissue was collected with informed patient consent and used according to Research Ethics Board at the Princess Margaret Cancer Centre, University Health Network approved protocols (06-196 and 15-9481). Upon receipt, a portion of tumor was fixed in 10%  buffer formalin for downstream histology, fragments were snap frozen and stored at −80 °C for genomic analyzes and the remaining tumor was minced and digested in 5–10 mL of Advanced DMEM/F12 containing 1X GlutaMAX, 10 mm HEPES and 1× antibiotic-antimycotic (ADF+++) with 500 µg/mL Liberase TH. Samples were incubated at 37 °C with gentle rocking on a nutator for 1 h. Samples were resuspended with a 5mL pipette to further dissociate undigested tissue, volume was brought up to 13 mL with ADF+++ and centrifugation was performed at 400 × *g* for 15 min, 4 °C. The cell pellet was resuspended in 2–5 mL TrypLE Express, triturated with a P1000 pipette and incubated at 37 °C for 15 min. Sample volume was then brought up to 13 mL with ADF+++ and passed over a 100 µm cell strainer. Centrifugation was performed at 300xg for 5 min, 4 °C. The cell pellet was treated with 1–2 mL Red Cell Lysing Solution for 5 min on ice; ADF+++ was added to bring volume to 10 mL and cells were pelleted at 300 × *g* for 5 min, 4 °C. Cells were counted and cell viability was determined by Trypan Blue staining. Next, 80,000 viable cells/well were plated in 50 µL/BME per well of a 24-well plate and were overlaid with 500 µL breast organoid media/well after allowing BME to gel for 10 min at 37 °C. 480,000 viable cells/well were plated in 300 µL 1 wt% EKGel/well of a 24-well plate and were overlaid with 750 µL breast organoid media/well after allowing EKGel with cells to solidify at 37 °C for at least 2 h.

### PDX tumor dissociation

PDX tumors were dissociated using the same as for patient tissue, except that 250 µg/mL Liberase TH was used and tissue was only digested for 45 min. An additional pellet washing step was included after the Liberase TH digestion and centrifugation and mouse cells were depleted from samples prior to plating in BME or EKGel, except where indicated in text. The gating strategy for flow cytometry is provided in Supplementary Fig. 15. For PDX growth, NSG mice (NOD.Cg NSG), Females at 4–6 wks of age, were housed in cages containing up to 5 animals on vented racks with 12/12 h light/dark cycle at 21–22 °C and 35–40% relative humidity. Ethics oversight was provided by the Research Ethics Board at the Princess Margaret Cancer Centre, University Health Network (UHN, #15-9481 and 06-196).

### Mouse cell depletion from PDX tumors

Digested PDX tumor cells were counted and resuspended in Depletion Buffer (PBS containing 0.5% BSA and 0.1× antibiotic-antimycotic). A mouse cell depletion was performed using Mouse Cell Depletion Cocktail, MACS LS columns and magnet as described by the manufacturer (Miltenyi). Cells were counted prior to and after depletion to determine depletion efficiency; this was also routinely monitored by flow cytometry. A fraction of cells were stained with 1:100 dilution FITC-anti-mouse-H-2K/H-2D (Clone 34-1-2S) and 1:100 dilution APC-anti-human-CD326 (EpCAM) prior to and after depletion to visualize mouse and human cell content. Flow cytometry was performed on a Canto II HTS instrument (BD Biosciences) and analysis was done using FlowJo software. The gating strategy for flow cytometry is shown in Supplementary Fig. 10.

### Organoid culture and passaging

Medium changes were performed every 3–4 days. Organoids were passaged every 2–3 weeks. For passage, medium was replaced with 1 mL TrypLE Express (Gibco)/well and the gels (BME or EKGel) were broken apart by manual shearing with a P1000. Organoids were incubated in TrypLE at 37 °C and triturated every 10 min for no more than an hour until dissociated to single cells. Cells were then centrifuged at 300 × *g* for 5 min, and the supernatant was removed. Cells were then suspended in either BME (50,000 viable cells/well were plated in 50 µL/BME per well) or in EKGel (300,000 viable cells/well were plated in 300 µL/EKGel per well) and plated in a 24-well-plate. In both matrices, the cell density was 1000 cells/µL. Once the matrix had solidified (10–15 min for BME and min 2 h for EKGel), the encapsulated cells were overlaid with media. Organoids were passaged every 2–3 weeks.

### Organoid and tissue histology

Organoids were removed from 24-well plates using a spatula and embedded in cryomolds using HistoGel (ThermoScientific). Once the organoid/HistoGel mix hardened, it was removed from the cryomold and placed in a histology cassette in 10% buffered formalin. Tissue pieces were also fixed in 10% formalin. Paraffin embedding and immunohistochemistry was performed by DDP-AMPL at the University Health Network. Antibody details and dilutions are included in Supplementary Table 4.

### Measurement of organoid diameter

Organoid diameter was measured from brightfield images in the ImageJ software (NIH). For each organoid, the diameter was determined as the geometric mean of two orthogonal diameters.

### Immunofluorescence staining

Eight-well chamber slides were coated with either BME or EKGel and left to solidify for at least 10 min or 2 h, respectively. Organoids were recovered from 24-well plates using Corning Cell Recovery Solution and plated in coated chamber slides in breast cancer organoid media (with 2% BME for those plated on wells coated in BME). Organoids were washed with PBS twice and then fixed in 5 wt% formalin for 30 min. Next organoids were washed three times with 400 µL 0.1 M glycine in PBS (10 min each wash). 400 µL of 0.5% Triton-X-100 in PBS was then added to permeabilize the cells for 5 min. The organoids were then washed three times (10 min each wash) with 400 µL immunofluorescence (IF) washing solution (IF wash) consisting of 0.05 wt. % NaN_3_, 0.1 wt. % Bovine Serum Albumin, 0.2 vol. % Triton-X-100 and 0.05 vol. % Tween-20 in PBS. Next, organoids were incubated with 400 mL of block solution (10 wt % goat serum in IF wash) for 90 min at room temperature. The block solution was replaced with 400 µL of the primary antibodies [1:800 anti-EpCAM(VU1D9); (Cell Signaling #2929) and 1:1000 anti-Ki67 (Abcam; ab15580)] in the block solution, and incubated overnight at room temperature. Organoids were then washed three times with IF wash (20 min each wash) and incubated with the secondary antibody solution consisted of 1:500 goat anti-rabbit Alexa Fluor 488 (Invitrogen^TM^) and 1:500 goat anti-mouse Alexa Fluor 568 (Invitrogen^TM^) in blocking solution for 60 min. The organoids were washed three times with 400 µL IF wash (20 min each wash). To stain for nuclei, 0.5 ng/mL of DAPI in PBS was added for 10 min at room temperature. The organoids were visualized using confocal microscopy (Zeiss LSM700 Confocal Microscope, Zeiss Zen software version 3.2).

### Drug assays

384-well clear-bottomed plates (Greiner) were pre-coated with 8 µL BME/well or 10 µL EKGel/well and left to solidify for at least 10 min and 2 h, respectively. Organoids were dissociated to single cells and 3000 cells were plated/well and left to grow for 4 days prior to the addition of equal volume of 2× drug. Cells were incubated with drugs for 5 days prior assay development using Cell Titer Glo 3D cell (Promega). AAC values were determined using PharmacoGx R package^83^.

### Establishing PDX from organoids

Organoids were dissociated to single cells using TrypLE Express and 1 million cells were injected into the mammary fat pad of NSG mice (NOD.Cg NSG), mice in a 100 µL volume (1:1 ratio of cells in organoid media: BME). Once tumors reached 150 mm^3^, they were treated with either vehicle control or taxol (20 mg/kg weekly). Tumor growth was monitored and recorded over time; once vehicle controls reached 1000–1500 mm^3^, all tumors were harvested and preserved for downstream characterization. NSG mice (NOD.Cg NSG), Females at 4–6wks of age, were used for PDX experiments. Mice were housed in cages containing up to 5 animals on vented racks with 12/12 hour light/dark cycle at 21–22 °C and 35–40% relative humidity. Ethics oversight was provided by the Research Ethics Board at the Princess Margaret Cancer Centre, University Health Network (UHN, #15-9481 and 06-196).

### RNA-Seq

Organoids were recovered from BME using 1mL Corning Cell Recovery media/well followed by a 1 h incubation on ice and centrifugation. Organoids were recovered from EKGel by resuspending in 1mL TrypLE Express/well, immediately adding ADF+++ and pelleted by centrifugation. RNA was isolated from recovered organoids and from snap frozen tissue fragments using NucleoSpin TriPrep. Library preparation was done using Illumina TruSeq Stranded Total RNA Sample Preparation kit with RiboZero Gold and samples were subjected to paired end sequencing (~80 million reads/sample) using the Illumina Novaseq 6000 at the Princess Margaret Genomics Centre. Gene expression profiles were generated using the Kallisto pipeline with GRCh38 as human reference^84^. Spearman correlation was used to measure the similarities between the different samples and DESeq2 R package was used to perform the gene expression differential analysis^85^. Pathway enrichment analysis was performed using Piano R package utilizing hypergeometric test^86^. Data and code to reproduce these analyses is available at https://github.com/bhklab/PDO_BME_EKGel.

### Statistical analysis

All data in the Results section are presented as mean ± st. dev., unless otherwise specified. Student’s *t*-test (Bonferroni-Dunn method, two-tailed) was used to determine statistical significance when comparing PDO diameters, doubling times, and drug AAC values for PDOs grown in EKGel and BME. Student’s *t*-test was performed in GraphPad Prism. The condition for statistical significance was *p* < 0.01. All micrographs are representative, and all microscopy experiments were repeated at least twice. For measurement of PDO diameter, *N* = 100 PDOs were measured per passage. For doubling time measurements, *N* = 4 biological replicates. For AAC measurements, *N* = 3 biological replicates.

### Reporting summary

Further information on research design is available in the Nature Research Reporting Summary linked to this article.

## Supplementary information

Supporting Information

Reporting Summary

## Data Availability

All other required for interpretation of the results is included in the Supplementary Information and manuscript or from the corresponding authors.

## Code availability

The computer code is open-source (Apache 2.0 License) and has been deposited on GitHub: https://github.com/bhklab/PDO_BME_EKGel.
