# Supplementary material for: Biomimetic hydrogel supports initiation and growth of patient-derived breast tumor organoids
Source: Nat Commun. 2022 Mar 18;13:1466. doi: 10.1038/s41467-022-28788-6 (PMC8933543; doi:10.1038/s41467-022-28788-6)
Supplement: Supplementary file 1 — Supporting Information [file 41467_2022_28788_MOESM1_ESM.docx]

**Supporting Information**

**Biomimetic hydrogel supports initiation and growth of patient-derived breast tumor** **organoids**

Elisabeth Prince^1^, Jennifer Cruickshank^2^, Wail Ba-Alawi^2,3^, Kelsey Hodgson^2^, Jillian Haight^2^, Chantal Tobin^2^, Drew Wakeman^2^, Alona Avoulov^1^, Valentina Topolskaia^1^, Mitchell J~~.~~ Elliott^2^, Alison P. McGuigan^4,5^, Hal K. Berman^2,6^, Benjamin Haibe-Kains^2,3^, David W. Cescon*^2^, Eugenia Kumacheva*^1,4,5^

^1^ Department of Chemistry, University of Toronto, 80 Saint George Street, Toronto, Ontario, Canada, M5S 3H6.

^2^ Princess Margaret Cancer Centre, 610 University Ave., Toronto, ON, Canada, M5G 2M9.

^3^ Department of Medical Biophysics, 101 College Street, University of Toronto, Toronto, ON, Canada, M5G 1L7.

^4^ Institute of Biomaterials and Biomedical Engineering, University of Toronto, 4 Taddle Creek Road, Toronto, Ontario, Canada, M5S 3G9.

^5^ Department of Chemical Engineering and Applied Chemistry, University of Toronto, 200 College Street, Toronto, Ontario, Canada, M5S 3E5.

^6^ Department of Laboratory Medicine and Pathobiology, University of Toronto, 1 King’s College Circle, Toronto, ON, Canada, M5S 1A8.

**
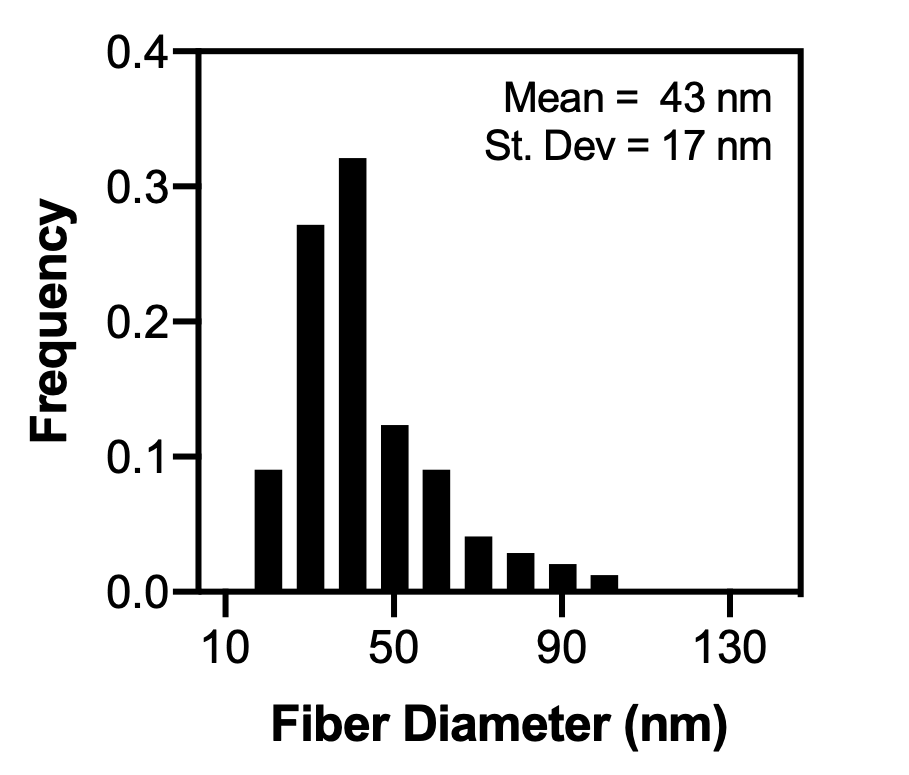
**

**Supplementary Figure 1. Histogram of fiber diameters in EKGel.** Fiber diameters measured from scanning electron microscopy images of EKGel using the ImageJ software. *N* = 200.

Supplementary Figure 1 shows a histogram of the fiber diameters in EKGel. Fiber diameters were measured from scanning electron microscopy images in the ImageJ software (NIH). The diameters ranged from 19 to 104 nm, with a mean and standard deviation of 43 and 17 nm, respectively. Notably, the width of a single CNC is 20 ± 4 nm, indicating that most fibers are composed of two or more CNCs packed side-by-side.

Supplementary Figure 2 shows strain and frequency sweeps of EKGel with 1 wt. % *a*-CNC and 2 wt. % gelatin at 37 °C. The regime of linear viscoelastic behavior is when the strain is between 0.8 and 50 %, and the frequency is between 0.1 and 30 Hz. Within this range, *G*’ is over 10-fold higher than *G*’’, indicating that EKGel is a stable hydrogel. For all other rheology experiments, the strain was kept at 1 % and frequency was kept at 1 Hz.


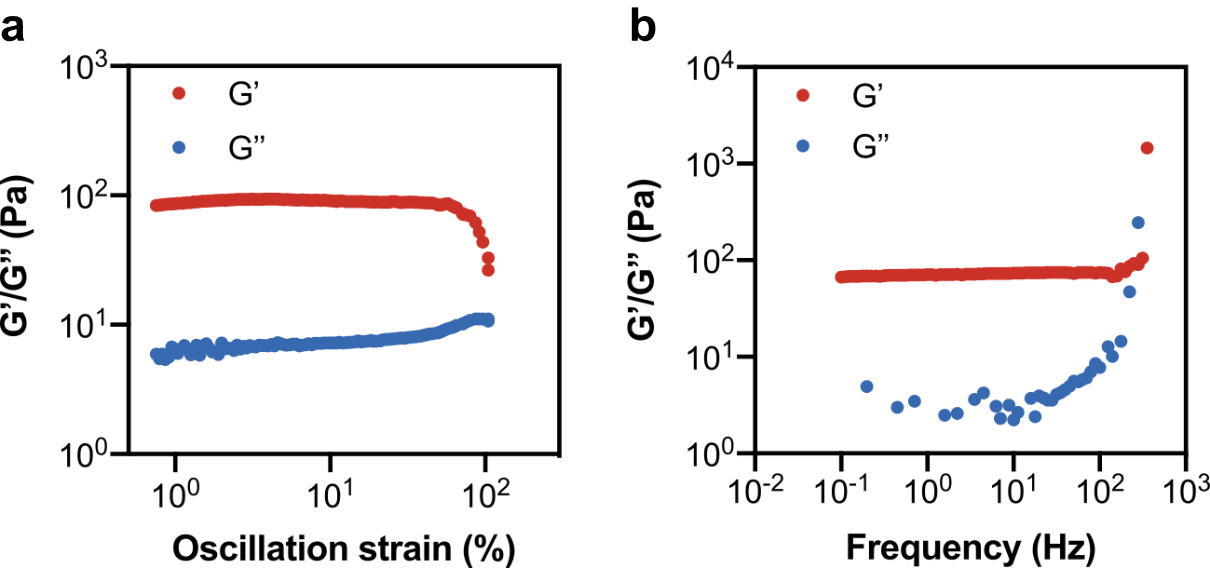


**Supplementary Figure 2. Frequency and strain dependence of EKGel’s rheological properties. a**, Frequency dependence of the storage (*G*’) and loss (*G*’’) modulus of EKGel at 1 % strain. **b**, Strain dependence of *G*’ and *G*’’ of EKGel at 1 Hz. In both (**a**) and (**b**) *C_a_*_-CNC_ was 1 wt. % and *C*_gelatin_ was 2 wt. %. were measured at 37 °C, and C. For all other rheological experiments, a frequency of 1 Hz and strain of 1 % was used, as this is within the linear viscoelastic regime.

Supplementary Figure 3 shows the cytocompatibility of EKGel with varying concentrations of *a*-CNC (*C_a_*_-CNC_). To evaluate the cytotoxicity of *a*-CNCs we cultured MCF-7 and MDA-MB-231 cells in EKGel hydrogels with different *C_a_*_-CNC_ and evaluated their normalized metabolic activity after 8 days of culture using the PrestoBlue assay. Metabolic activity was defined as the fluorescence intensity resazurin (excitation and emission wavelengths of 550 and 596 nm, respectively) on day 8, divided by the average fluorescence on day 0. Supplementary Figure 3 shows that for MDA-MB-231 cells, at all concentrations of *a*-CNC the normalized metabolic activity was greater than 1. This result indicated that the metabolic activity increased from day 0 to day 8, the cells proliferated and thus the *a*-CNCs were not cytotoxic. For MCF-7 cells, the normalized metabolic activity was greater than 1 for all concentrations, with the exception of *C_a-_*_CNC_ = 2.5 wt%, where the normalized metabolic activity was 0.45, however the metabolic activity at C*_a_*_-CNC_ = 2.5 wt% could decrease because of the increased stiffness of the hydrogel.


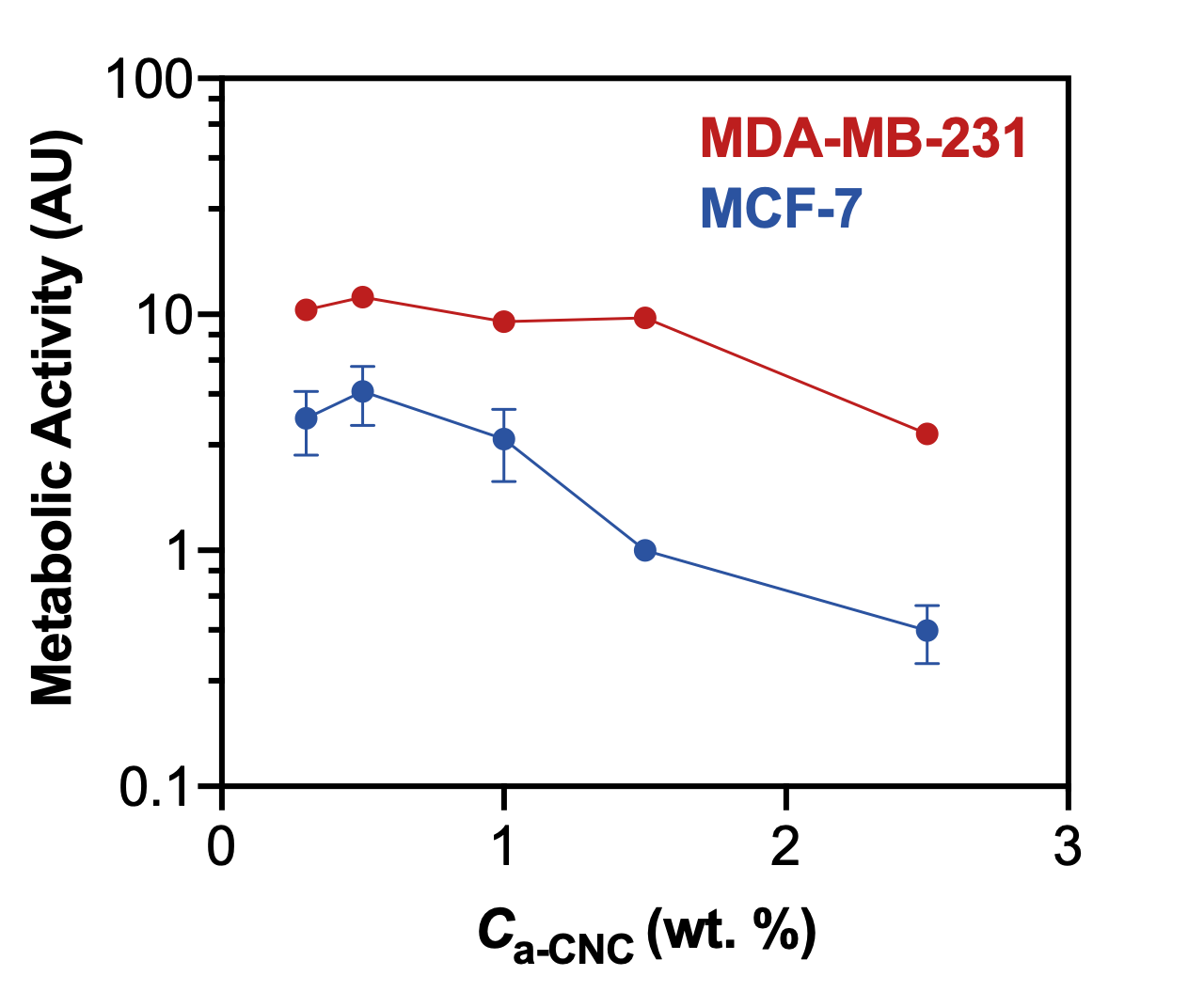


**Supplementary Figure 3. Cytocompatibility of EKGel with different *C_a_*_-CNC_.** Normalized metabolic activity of MCF-7 and MDA-MB-231 at different *C_a_*_-CNC_ with constant *C*_gelatin_ = 2.0 wt%. Metabolic activity was determined after 8 days of culture using the PrestoBlue assay. Data shown as Mean ± St. Dev (*N* = 3). Some error bars are smaller than the symbol and do not appear.

Supplementary Figure 4 shows the geometry of the microfluidic device used to examine hydrogel stability under flow conditions. The device consists of an array of cylindrical 100 µm-diameter microwells that are connected with a common fluid supplying channel, The devices were fabricated using soft lithography[^87^](https://paperpile.com/c/UCRtor/yi41).

Uniformly sized droplets of liquid EKGel and BME precursors were formed in the microwells using a self-digitization method reported elsewhere[^61,88^](https://paperpile.com/c/UCRtor/q5Ce+PqXU). Following the formation of droplets, the MF device was incubated at 37 °C for 1-2 hours to transform droplets into microgels. The medium was then continuously perfused through the supplying channel at *v*_medium_ = 96 µm/s, that is, close-to-physiological interstitial flow velocities and the flow velocities used in microfluidic organoid on a chip platforms[^77,89–92^](https://paperpile.com/c/UCRtor/4UUi+o1L7+O3EK+Yl27+xQdG). Flow rate was controlled by Harvard Apparatus syringe pump)

Brightfield microscopy images of the microgels in the microwells were taken at different time intervals and the average microgel volume was determined by measuring the average cross-sectional area of the microgels using ImageJ software (NIH), and calculating their volume according to equation S1.

| $v=\frac{{area}^{3/2}}{\sqrt{\pi}}$ | (S1) |
| --- | --- |

The volume was normalized to the initial volume, *v*_0_, of the microgel before perfusion of the medium. Supplementary Figure 4 shows the images of the BME and EKGel microgels before and after flow.

To confirm that the shear stress imposed by the flow of liquid governs BME degradation, we also measured change in the normalized volume of BME and EKGel microgels over time in the absence of flow. While there was a small reduction in the hydrogel volume over 144 hours (11 and 6% volume reduction for BME and EKGel, respectively), this decrease in volume was significantly larger for BME, when the medium was perfused at 96 µm/s through the supplying channel (Figure 1d, Main Text).


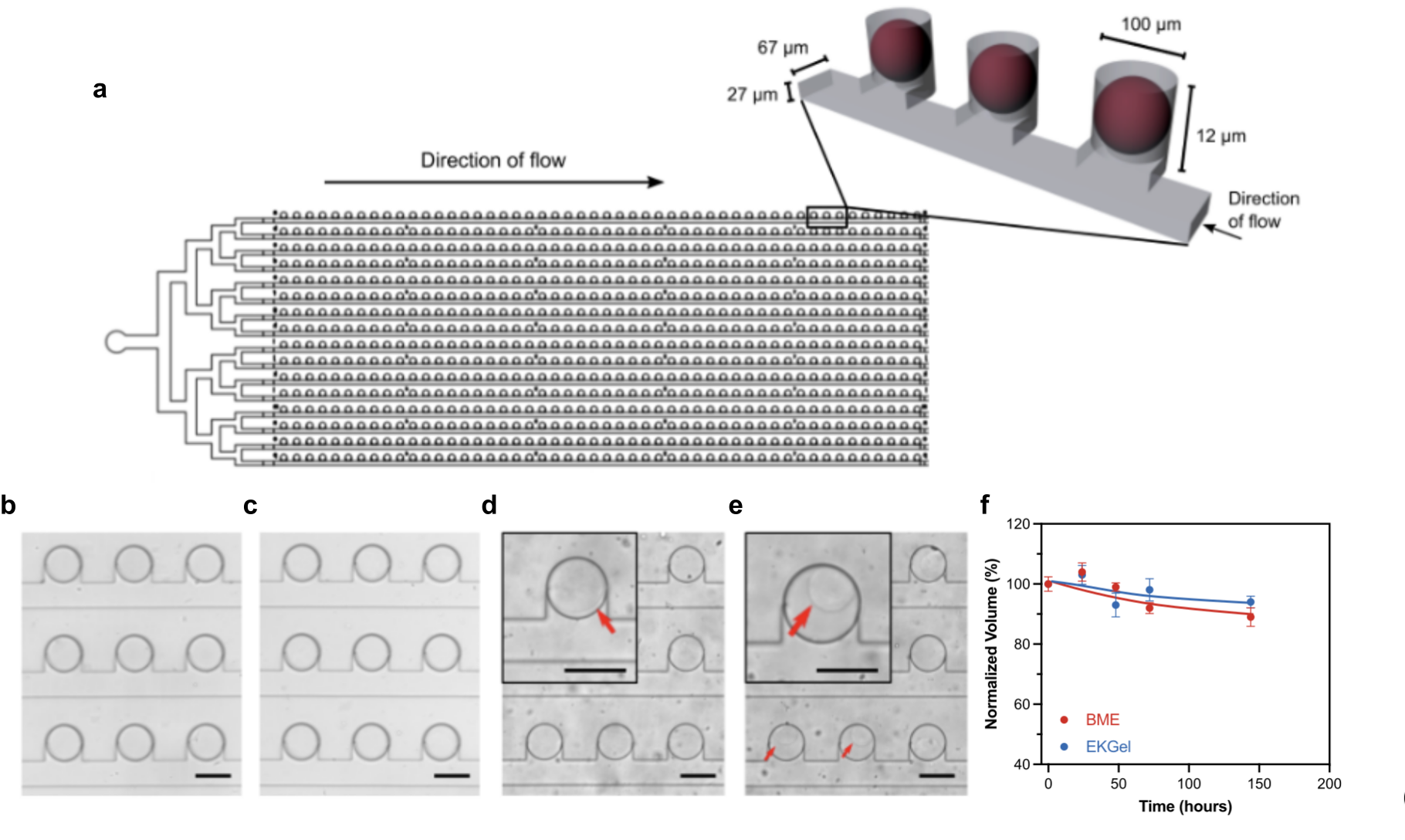


**Supplementary Figure 4.** Microfluidic device for examination of microgel stability (fabricated by FlowJEM, Inc). **a**, Design of the microfluidic device. Inset: dimensions of the supplying channel and microwells. **b**, **c**, Images of EKGel (b) and BME (c) microgels on day 0. **d, e,** Images of EKGel and BME microgels after 6 days of perfusion of the cell culture media through the supply channel at *v*_media_ = 96 µm/s. Insets in (d, e) show high-magnification images, red arrows indicate the edges of the microgels. **f**, Reduction in EKGel and BME volume in the absence of flow. Lines are added for eye-guidance, data shown as mean ± st. dev of *N* = 50 microgels in a single experiment.

To qualitatively investigate possible differences in matrix adhesion or cytoskeleton organization between BME and EKGel, we performed staining for focal adhesion kinase (FAK). Line 3 organoids were stained with human FAK rabbit polyclonal antibody (Proteintech, 12636-1-AP) to visualize focal adhesions (Supplementary Figure 5). Qualitatively, we observed no difference in FAK staining between the two matrices. We also stained actin filaments with Alexa Fluor 546 phalloidin to investigate whether any differences in matrix adhesions resulted in a change in cytoskeleton structure. In Supplementary Figure 5, we show that no difference was observed in cytoskeleton structure between BME and EKGel.


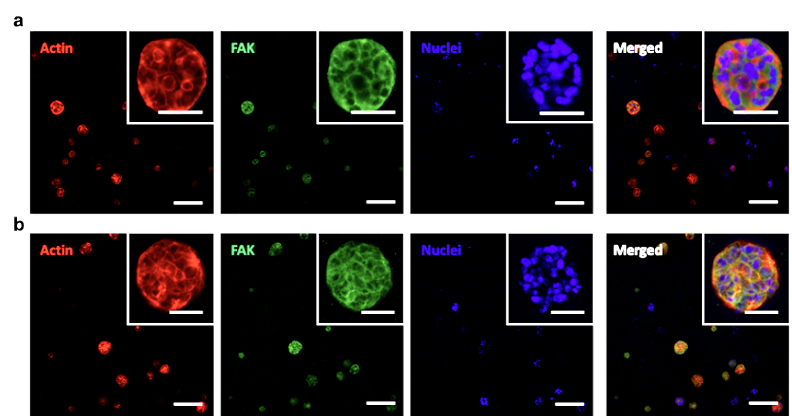


**Supplementary Figure 5.** FAK and actin staining of PDO-3 organoids in EKGel and BME. Images of PDO-3 organoids grown in EKGel **a**, and BME **b**, and stained with Alexa Fluor 546 phalloidin (red), human FAK rabbit polyclonal antibody (green) and DAPI (blue). Right most images are the merged images of all channels. Insets show higher magnification images. Scale bars are 200 µm, and 50 µm in insets.

**
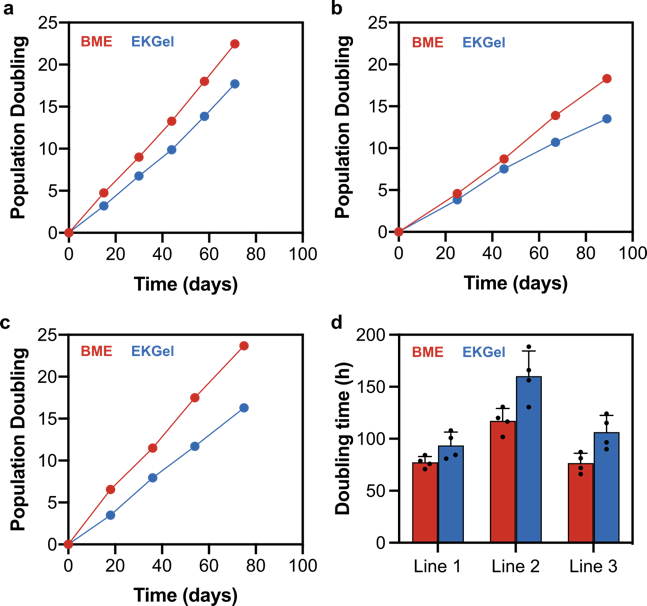
**

**Supplementary Figure 6. Population doubling of breast patient derived tumour organoids (PDOs) grown in EKGel and BME. a-c**, Number of population doublings over time in both EKGel and BME for (**a**) PDO-1, (**b**) PDXO-2, and (**c**) PDO-3. **d**, The doubling times of breast PDOs in BME and EKGel. Data shown as mean ± st. dev (*N* = 4). While the doubling times are longer in EKGel then in BME for all three lines, the differences are not statistically significant (Student’s t-test, Bonferroni-Dunn method, two-tailed, *p*>0.01).

Supplementary Figure 6 shows the population doublings over time for lines 1 (Fig. S6a), 2 (Fig. S6b) and 3 (Fig. S6c). The number of population doublings was determined by counting the number of total number cells after dissociation of the organoids at each passage. The dissociated cells were counted using a hemocytometer. In all three lines the rate of cell proliferation was slightly higher in BME than in EKGel. Similarly, the doubling times for cells from all three organoid lines grown in BME were shorter than those grown in EKGel, however the differences are not statistically significant Student’s t-test, Bonferroni-Dunn method, two-tailed, *p*>0.01).


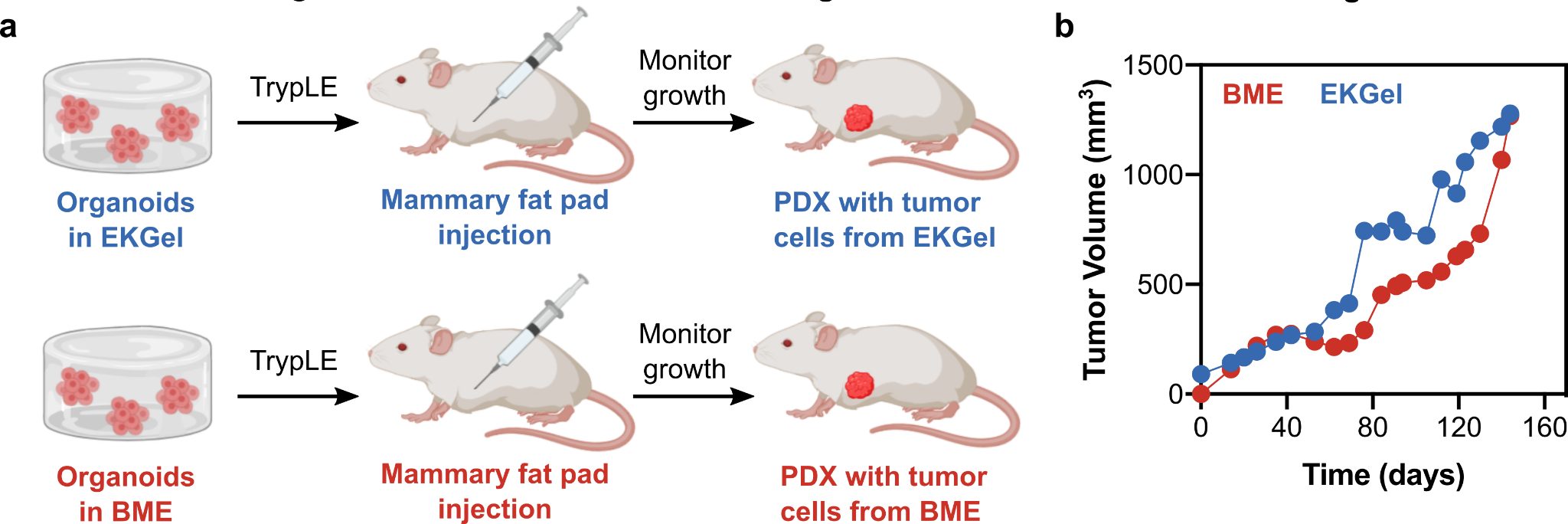


**Supplementary Figure 7. In vivo engraftment of organoids from EKGel and BME.** (**a**), Schematic of *in vivo* xenograft generation. Organoid-derived xenografts are generated from tumor cells isolated from PDOs formed in BME or EKGel. (**b**), Growth of tumor volume over time from PDXOs generated in BME (red) and EKGel (blue).

To verify that organoid growth in EKGel does not influence the tumor initiating capability of the breast cancer cells, we initiated xenografts from PDOs maintained in each matrix in immunocompromised mice (Supplementary Figure 7a). PDXO-2 (ER-/PR-/HER2-) organoids grown in EKGel and BME were enzymatically dissociated, and 1 million isolated cells were injected into the mammary fat pad of non-obese diabetic (NOD)/SCID mice. Tumor growth initiated from the cells obtained from organoids grown in both EKGel and BME, and tumor size endpoints were achieved in similar timeframes, indicating that organoid growth in either matrix did not compromise the tumorigenicity or long-term growth potential of patient-derived breast cancer cells. Tumors derived from organoids grown in EKGel and BME grew at similar rates, and both grew to a final volume of 1.3 cm^3^ by 144 days post-injection (Supplementary Figure 7b).


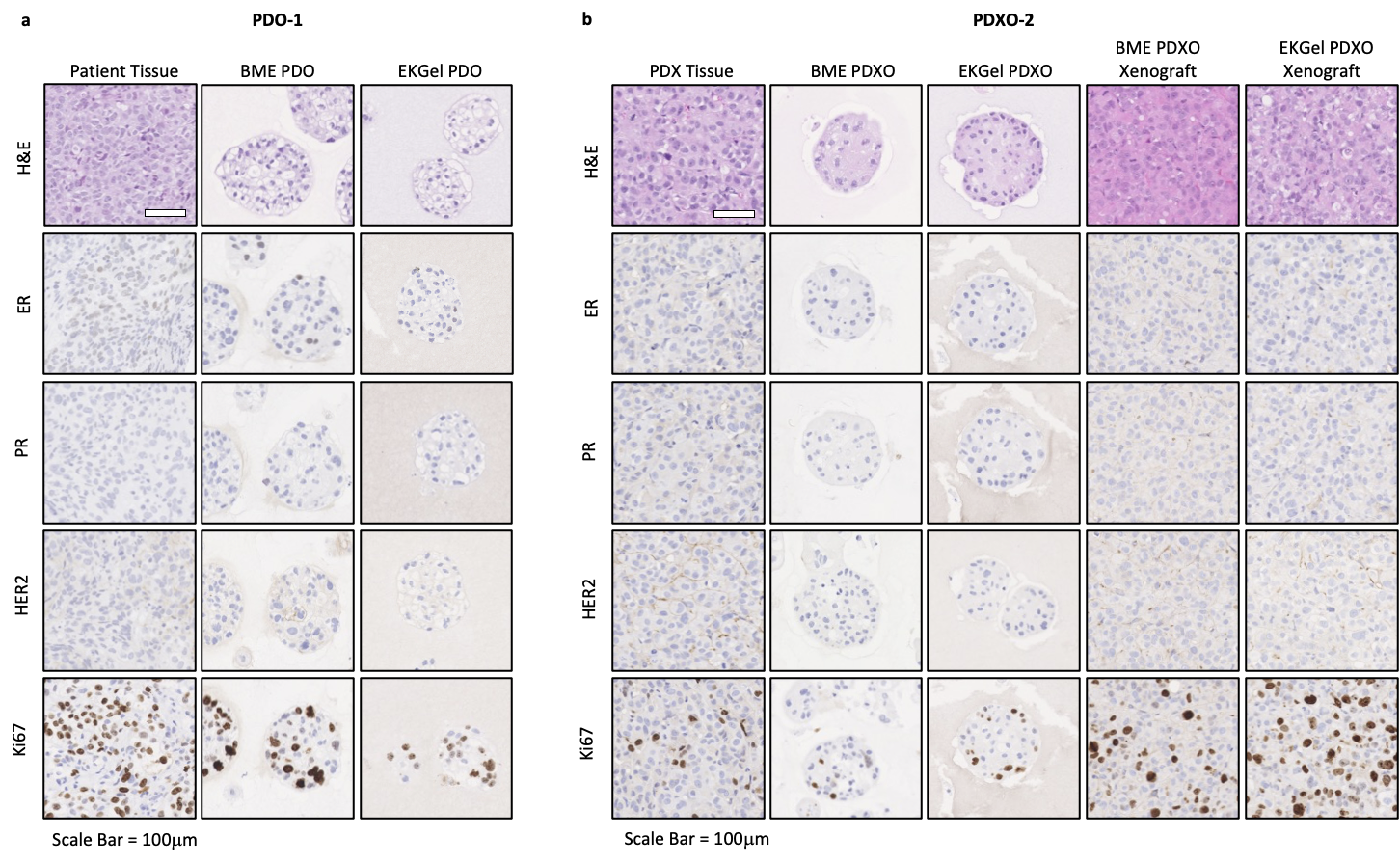


**Supplementary Figure 8. Immunohistological staining of Lines 1 and 2.** **a**, From left to right shows staining of the source PDX tissue, PDXOs in BME and EKGel, as well as PDXO derived xenografts from PDXOs in BME and EKGel. **b**, From left to right shows staining of the source patient tissue, and PDOs in BME and EKGel.

Supplementary Figure 9 shows all of the dose response curves to anticancer drugs for organoids grown in BME and EKGel for lines 1 (Fig. S9a-c), 2 (Fig. S9d-f), and 3 (Fig. S9g-i). The dose response curves were generated using the CellTiter Glo assay, as described in the methods and materials section.


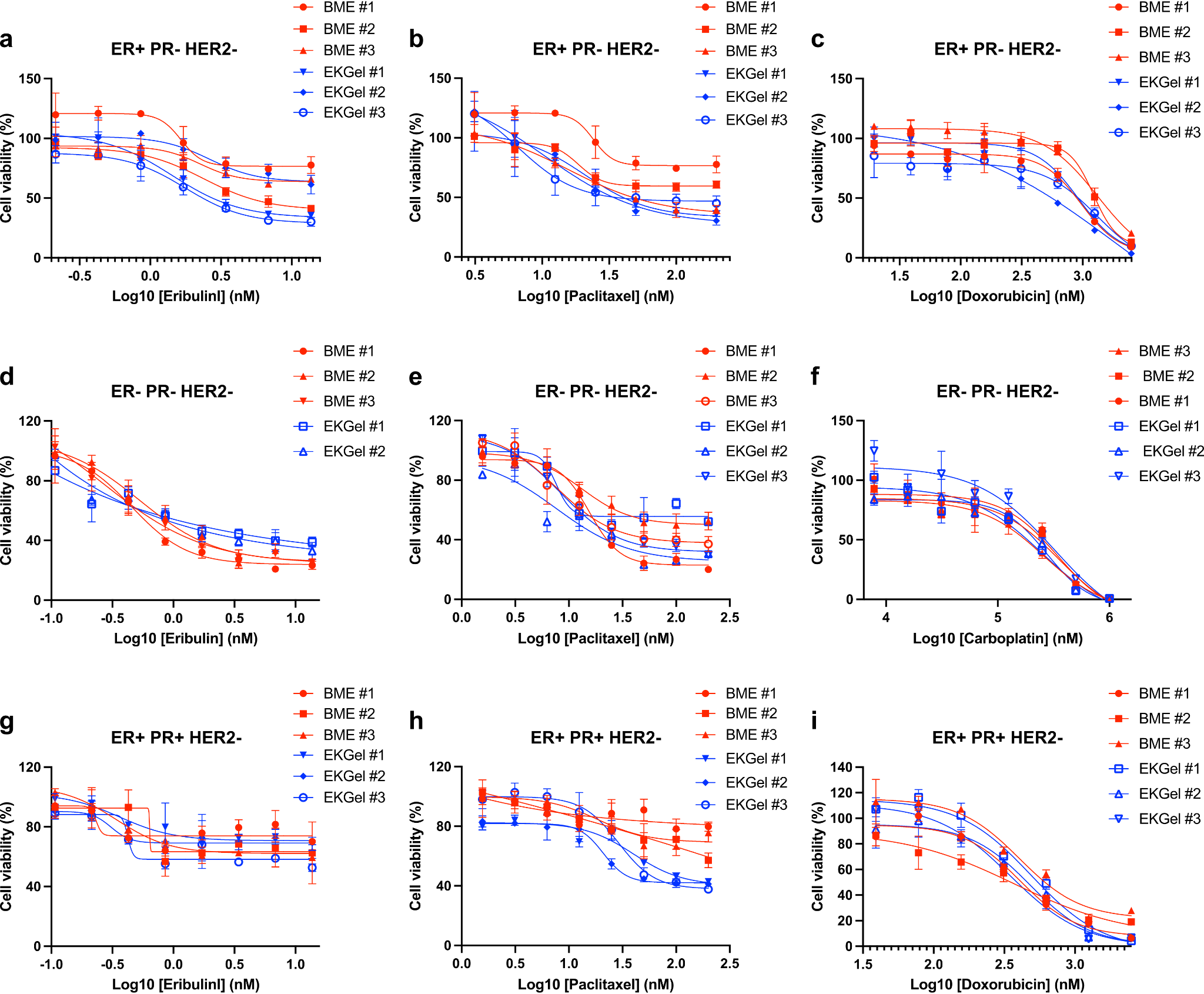


**Supplementary Figure 9. Dose response curves for breast PDOs to different drugs.** **a-c**, Response of PDO-1 to eribulin (**a**), paclitaxel (**b**), and doxorubicin (**c**). **d-f**, Response of PDXO-2 to eribulin (**d**), paclitaxel (**e**), and carboplatin (**d**). **g-i**, Response of PDO-3 to eribulin (**g**), paclitaxel (**h**), and doxorubicin (**i**). Each line represents a separate biological trial. Data shown as mean ± st. dev (*N* = 3 technical replicates).

To evaluate whether PDO growth in EKGel impacts their chemosensitivity in vivo, the organoids grown in EKGel and BME from PDXO-2 (ER-/PR-/HER2-) cells were enzymatically digested by TrypLE Express (Gibco) and the cells were implanted into the mammary fat pad of non-obese diabetic (NOD)/SCID mice to generate xenografts, which were subsequently treated with paclitaxel; Supplementary Figure 10A shows a schematic illustrating this experiment. Once the tumors were established and reached a volume of ~150 mm^3^, paclitaxel treatment was initiated and delivered intravenously at an established and clinically relevant dose (20 mg/kg), on a weekly schedule. The animals were sacrificed when the tumors reached the humane endpoint (1500 mm^3^). Supplementary Figures 10B and C show the growth of tumors with and without drug administration in EKGel (EKGel-tumors) and BME (BME-tumors) respectively. The untreated controls grew at similar rates in both EKGel and BME. Both the EKGel-tumors and BME-tumors showed response to paclitaxel, with substantial tumor growth inhibition, but not regression observed in the drug-treated tumors. Furthermore, the growth rate of the paclitaxel-treated EKGel-tumors and BME-tumors were similar, indicating that the *in vivo* drug response appears unimpacted by organoid growth in EKGel *vs*. BME.


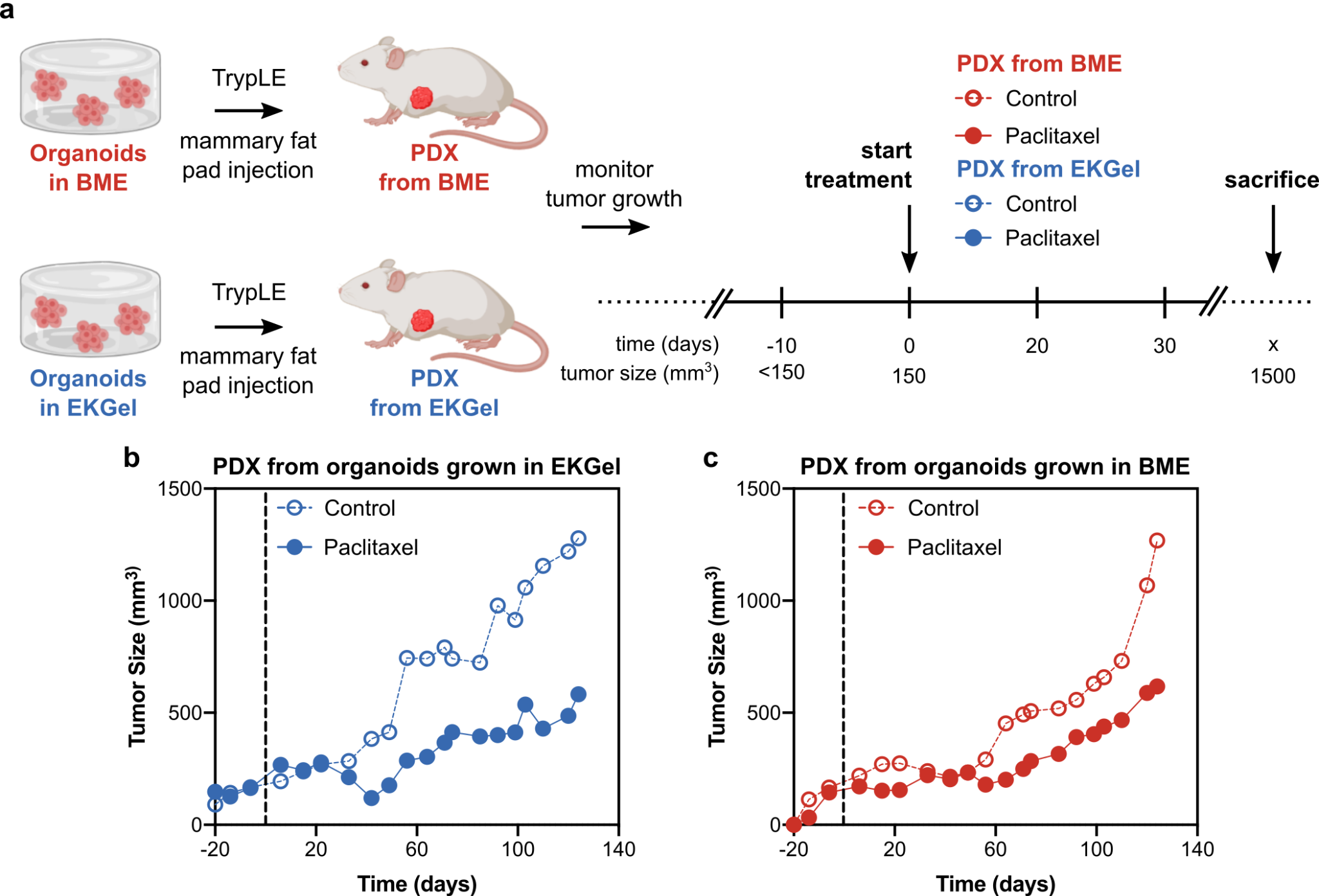


**Supplementary Figure 10. Xenograft drug response data. a**, Schematics of experiments evaluating *in vivo* drug response of cells obtained from PDXO-2. Cells from organoids grown in BME and EKGel were isolated and orthotopically transplanted into mice. Once tumor volume exceeded 150 mm^3^, the PDXs were treated with either paclitaxel, or water (control). Mice were sacrificed when tumor volume exceeded 1000 mm^3^. **b**, **c**, Variation in tumor volume over time for PDXs treated with paclitaxel or control. In (b) and (c), *N* = 1, in accordance with established protocols[^93^](https://paperpile.com/c/UCRtor/GSoW). The PDX were derived from organoids grown in EKGel (b) and BME (c).

Supplementary Figure 11 shows immunofluorescence staining of a PDX sample plated in both EKGel and BME for Human EpCAM and mouse H2K-d. The sample plated in BME contained both human patient-derived organoids (red, human EpCAM positive) and large clusters of mouse epidermal cells (green, mouse H2K-d positive). In addition, both single mouse and human cells were observed. In EKGel, only human organoids were observed, while no large clusters of mouse cells were observed. There were, however, single H2K-d positive mouse cells present (shown with the yellow arrows) indicating that viable mouse epidermal cells were present in EKGel, but unlike in BME, they did not proliferate and overtake the human cells.


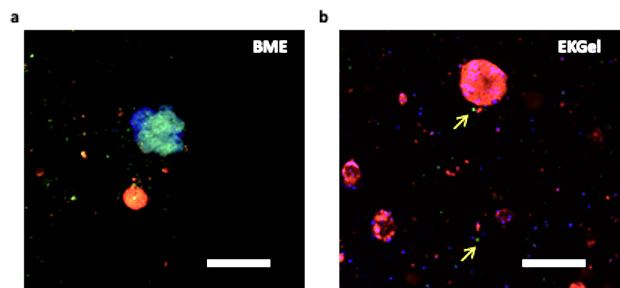


**Supplementary Figure 11. Immunofluorescence staining of a PDX samples plated in** **a**, BME and **b**, EKGel, stained for mouse H2K-d (green), human EpCAM (red), and nuclei (blue). Green clusters are colonies of contaminating mouse cells. Red organoids are human patient-derived tumor organoids. Single mouse cells in EKGel are indicated with yellow arrows. Scale bars are 200 µm.

Supplementary Figure 12 shows the growth of cells isolated from a mouse mammary gland in both BME and EKGel (using mouse mammary gland organoid media[^16^](https://paperpile.com/c/UCRtor/82n9)) over seven days. Significantly stronger growth of mouse mammary cells was observed in BME, in comparison with EKGel.


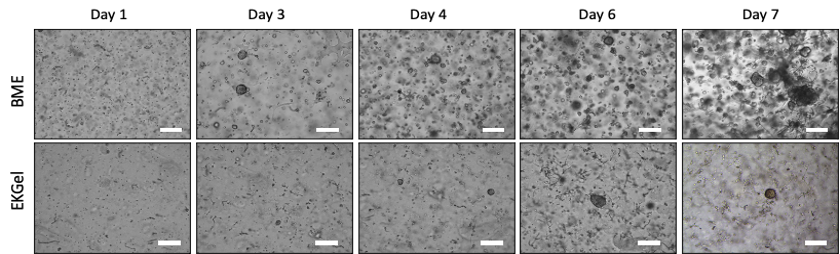


**Supplementary Figure 12.** Growth of cells isolated from the mammary gland of non-obese diabetic (NOD)/SCID mice cultured in BME (top row) and EKGel (bottom row) over one week. Scale bars are 100 µm.

**
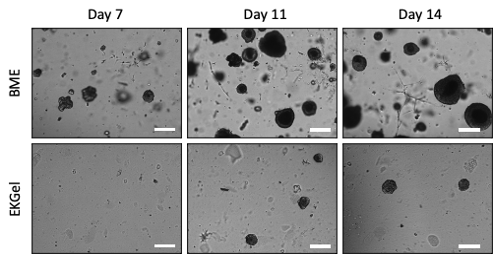
**

**Supplementary Figure 13.** Growth of cells isolated from the skin of digested of non-obese diabetic (NOD)/SCID mice cultured in BME (top row) and EKGel (bottom row) over one week. Scale bars are 100 µm.

Similarly, Supplementary Figure 13 shows the growth of cells isolated from mouse skin in BME and EKGel. In BME, there was extensive proliferation of the skin cells, and several large dark clusters of mouse cells formed. In contrast, while some colonies of mouse skin cells formed in the skin sample plated in EKGel (indicating that EKGel is not cytotoxic to the skin cells), the density of cells was significantly lower and the colonies were notably smaller, indicating that the proliferation is suppressed in EKGel relative to BME. We note that the morphology of the colonies observed in BME in Figure 2 were similar to that of the large clusters of the contaminating mouse cells that we observed in PDXO samples plated in BME (Main Text, Figure 6). Both were large, dark clusters with a visible core. Furthermore, the morphology of these clusters was similar to the morphology of mouse epidermal organoids[^94^](https://paperpile.com/c/UCRtor/XJsF) that are prepared by digesting mouse skin samples in TrypLE and plating them in BME in organoid media.


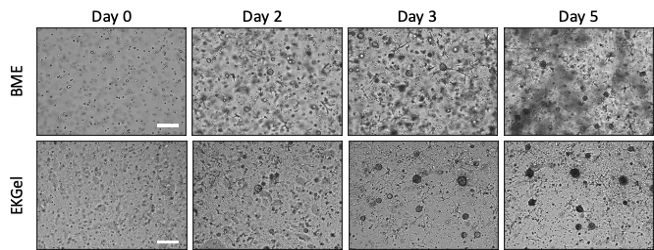


**Supplementary Figure 14.** Growth of KBP mouse mammary tumor cells in BME (top row) and EKGel (bottom row) over one five days. Scale bars are 100 µm.

Supplementary Figure 14 shows the results of culture KPB mouse mammary tumor cells in EKGel and BME, which should escape dependence on the residual growth factors in BME, and thus show similar proliferation in BME and EKGel. As expected, KPB mouse mammary tumor cells proliferated at a similar rate in BME and EKGel.


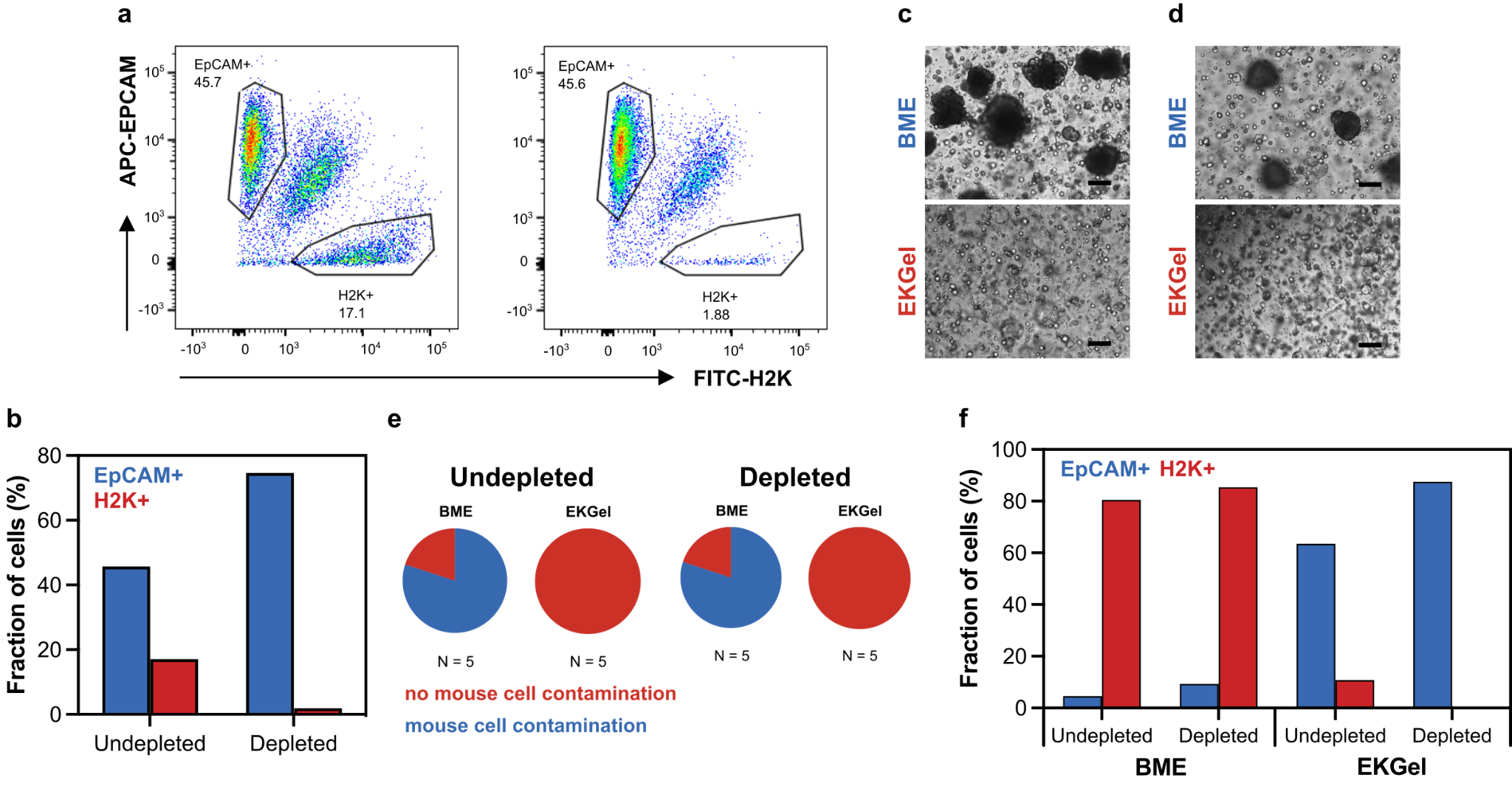


**Supplementary Figure 15. Initiating organoids directly in BME or EKGel without mouse cell depletion. (a**) Flow cytometry profile of a dissociated PDX tumor with (right panel) or without (left panel) mouse cell depletion. (**b**) Quantification of the percentage murine H2K+ cells and human EpCAM cells in the undepleted and mouse cell depleted sample as determined by flow cytometry in (a). (**c**) Brightfield microscopy images of undepleted PDX cells 14 days after plating in BME or EKGel in parallel. (**d**) Brightfield microscopy images of mouse cell-depleted PDX sample from the same tumor plated in BME or EKGel in parallel after 14 days in culture. Scale bards in (c, d) are 100 µm. (**e**) Five independent PDX tumours were processed for organoids with and without mouse cell depletion and plated in BME and EKGel and monitored for mouse cell contamination. (**f**) Quantification of the percentage of H2K+ and EpCAM+ cells in undepleted versus depleted cells shown in (c, d) as determined by flow cytometry.


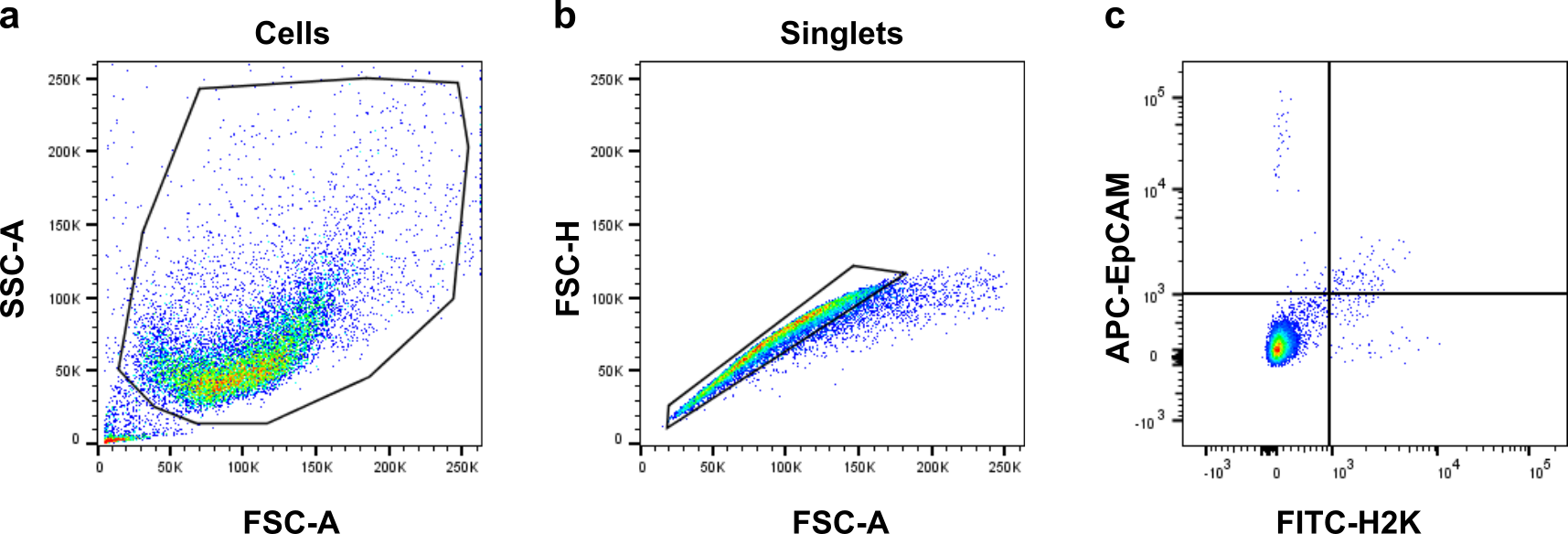


**Supplementary Figure 16. Flow cytometry gating strategy.** (**a**) Cells were selected using SSC-A/FSC-A, followed by singlets selection using FSC-H/FSC-A (**b**). (**c**) FITC-H2K and APC-EpCAM were used to gate for mouse and human cells, respectively.

**Supplementary Table 1. Components of breast organoid media.**

| **Medium Component** | **Final Concentration** | **Supplier** | **Catalogue No.** |
| --- | --- | --- | --- |
| A83-01 |  | Tocris | 2939 |
| Advanced DMEM/F12 | 1X | Thermo Fisher Scientific | 12634-010 |
| Antibiotic-antimycotic (100X) | 1X | Thermo Fisher Scientific | 15240-062 |
| B27 Supplement (50X) | 1X | Thermo Fisher Scientific | 17504-044 |
| EGF | 5 ng/mL | Fisher | CB-40052 |
| FGF7 | 5 ng/mL | Peprotech | 100-19 |
| FGF10 | 20 ng/mL | Peprotech | 100-26 |
| GlutaMAX (100X) | 1X | Thermo Fisher Scientific | 35050-061 |
| HEPES (1M) | 10 mM | Thermo Fisher Scientific | 15630-080 |
| N-Acetyl-L-cysteine | 1.25 mM | Sigma | A9165 |
| Neuregulin-1 | 5 mM | Peprotech | 100-03 |
| Nicotinamide | 5 mM | Sigma | N0636 |
| Noggin | 100 ng/mL | Peprotech | 120-10C |
| R-Spondin-3 (or R-Spondin 1 Conditioned Medium) | 250 ng/mL (or 10% Conditioned Medium) | Peprotech | 120-44 |
| SB202190 | 500 nM | Sigma | S7067 |
| Y-27632 | 5 mM | Sigma | S1049 |

**Supplementary Table 2. Details for PDXOs and PDOs grown in EKGel and BME.**

| **Line** | **Sex** | **Menopausal Status** | **Metastatic Status** | **Histology** | **Tissue Origin** | **Tissue Source** | **Prior Treatments Received** |
| --- | --- | --- | --- | --- | --- | --- | --- |
| **1** | Female | Peri-menopausal | Non-Metastatic | IDC | Primary Breast | Breast | fluorouracil, epirubicin, cyclophosphamide, docetaxel |
| **2** | Female | Post-menopausal | Metastatic | IDC | Metastatic site | Cutaneous Metastasis | doxorubicin, cyclophosphamide, docetaxel, aromatase inhibitor, pembrolizumab |
| **3** | Female | Pre-menopausal | Non-Metastatic | IDC | Primary Breast | Breast | none |
| **Line** | **ER** | **ER%** | **PR** | **PR%** | **HER2** | **HER2 IHC** | **HER2 FISH** |
| **1** | positive | 5-10% | negative | 0% | negative | 0 | N/A |
| **2** | negative | 0% | negative | 0% | negative | 0 | N/A |
| **3** | positive | 81-90% | positive | 21-30% | negative | 1+ | N/A |

**Supplementary Table 3. List of all reagents.**

| **Commercial Kits** | | |
| --- | --- | --- |
| **Kit Name** | **Supplier** | **Catalogue No.** |
| Cell Titer Glo 3D | Promega | PRG9683 |
| MACS LS Columns | Miltenyi | 130-042-401 |
| Mouse Cell Depletion Kit | Miltenyi | 130-104694 |
| NucleoSpin TriPrep, Mini Kit | Magerey-Nagel | 740966.50 |
| **Other Reagents** | | |
| **Reagent Name** | **Supplier** | **Catalogue No.** |
| Carboplatin | UHN Pharmacy | N/A |
| Corning Cell Recovery Solution | Fisher | CACB354253 |
| Cultrex Reduced Growth Factor Basement Membrane Extract, Type 2 | Biotechne | 3533-010-02 |
| InSolution Doxorubicin, Hydrochloride | Calbiochem | CAS 25316-40-9 |
| Eribulin | UHN Pharmacy | N/A |
| TrypLE Express | ThermoFisher Scientific | 1265028 |

**Supplementary Table 4. List of antibodies.**

| **Antibodies** | | | |
| --- | --- | --- | --- |
| **Antibody Name** | **Supplier** | **Catalogue No.** | **Dilution Used (Application)** |
| FITC-anti-mouse H-2K/H-2D (Clone 34-1-2S) | Biolegend | 114706 | 1:100 (Flow Cytometry) |
| APC anti-human CD326 (EpCAM) Antibody | Biolegend | 324208 | 1:100 (Flow Cytometry) |
| EpCAM (VU1D9) | Cell Signaling Technologies | 2929 | 1:800 (IF) |
| Ki67 | Abcam | ab15580 | 1:1000 (IF) |
| ER | Abcam | ab16660 | 1:200 (IHC) |
| PR | Dako | M3569 | 1:200 (IHC) |
| HER2 | Ventana/Roche | 790-4493 | No dilution (pre-diluted) (IHC) |
| MIB1/Ki67 | Dako | M7240 | 1:100 (IHC) |
