# Supplementary material for: Biomimetic hydrogel supports initiation and growth of patient-derived breast tumor organoids
Source: Nat Commun. 2022 Mar 18;13:1466. doi: 10.1038/s41467-022-28788-6 (PMC8933543; doi:10.1038/s41467-022-28788-6)
Supplement: Supplementary file 2 — Reporting Summary [file 41467_2022_28788_MOESM2_ESM.pdf]

nature research

Corresponding author(s): David W. Cescon, Eugenia Kumacheva
Last updated by author(s): Jan 10, 2022

Reporting Summary

Nature Research wishes to improve the reproducibility of the work that we publish. This form provides structure for consistency and transparency in reporting. For further information on Nature Research policies, see our Editorial Policies and the Editorial Policy Checklist.

Statistics

For all statistical analyses, confirm that the following items are present in the figure legend, table legend, main text, or Methods section.

n/a	Confirmed	
☐	☒	The exact sample size (n) for each experimental group/condition, given as a discrete number and unit of measurement
☐	☒	A statement on whether measurements were taken from distinct samples or whether the same sample was measured repeatedly
☐	☒	The statistical test(s) used AND whether they are one- or two-sided<br>*Only common tests should be described solely by name; describe more complex techniques in the Methods section.*
☒	☐	A description of all covariates tested
☐	☒	A description of any assumptions or corrections, such as tests of normality and adjustment for multiple comparisons
☐	☒	A full description of the statistical parameters including central tendency (e.g. means) or other basic estimates (e.g. regression coefficient) AND variation (e.g. standard deviation) or associated estimates of uncertainty (e.g. confidence intervals)
☐	☒	For null hypothesis testing, the test statistic (e.g. F, t, r) with confidence intervals, effect sizes, degrees of freedom and P value noted<br>*Give P values as exact values whenever suitable.*
☒	☐	For Bayesian analysis, information on the choice of priors and Markov chain Monte Carlo settings
☒	☐	For hierarchical and complex designs, identification of the appropriate level for tests and full reporting of outcomes
☒	☐	Estimates of effect sizes (e.g. Cohen's d, Pearson's r), indicating how they were calculated

Our web collection on statistics for biologists contains articles on many of the points above.

Software and code

Policy information about availability of computer code

Data collection	Zeiss Zen software (version 3.2) was used for confocal microscopy.
Data analysis	GraphPad prism (version 8) was used to generate graphs, determine IC50s, and perform statistical analysis.<br>IImageJ (NIH, version 1.52q) was used for image analysis to measure organoid diameter.<br>Pathway enrichment analysis was performed using Piano R package utilizing hypergeometric test.<br>Flow cytometry analysis was performed using the FlowJo software.<br>DESeq2 R package was used to perform the gene expression differential analysis. Data and code to reproduce these analyses is available at https://github.com/bhklab/.

For manuscripts utilizing custom algorithms or software that are central to the research but not yet described in published literature, software must be made available to editors and reviewers. We strongly encourage code deposition in a community repository (e.g. GitHub). See the Nature Research guidelines for submitting code & software for further information.

Data

Policy information about availability of data

All manuscripts must include a data availability statement. This statement should provide the following information, where applicable:
- Accession codes, unique identifiers, or web links for publicly available datasets
- A list of figures that have associated raw data
- A description of any restrictions on data availability

For RNAseq analysis, the data and code to reproduce the analyses are publicly available at https://github.com/bhklab/. All other data is either included in the manuscript or will be made available upon request.

Field-specific reporting

Please select the one below that is the best fit for your research. If you are not sure, read the appropriate sections before making your selection.

- [x] Life sciences
- [ ] Behavioural & social sciences
- [ ] Ecological, evolutionary & environmental sciences

For a reference copy of the document with all sections, see nature.com/documents/nr-reporting-summary-flat.pdf

Life sciences study design

All studies must disclose on these points even when the disclosure is negative.

Sample size	The number of PDO and PDXO lines used in this study was determined by the availability of primary breast tumor tissue and PDX tumors. A total of 22 patient-derived organoid lines were initiated in EKGel and BME (17 derived from PDX tumors, and 5 derived from primary patient tumors).<br><br>Two PDO lines and one PDXO line, each with different receptor statuses, were selected to compare growth and phenotype in both matrices in detail. The three different lines were selected to evaluate if EKGel can support the culture of organoids with different tissue sources and hormone receptor statuses.
Data exclusions	Drug assays where the EKGel or BME hydrogels were torn or visibly broken apart, which would change the cell count per well, were excluded from the analysis. No other data was excluded from this study.
Replication	All experiments were replicated and the number of replications for each experiment is reported in the main text.
Randomization	The PDO and PDXO samples were not organized into experimental and control groups, and thus randomization was not applicable.<br>For PDX experiments for in-vivo drug response, the mice were randomly allocated into treated and untreated groups.
Blinding	Blinding was performed for analysis of organoid diameters. The persons analyzing the images were unaware of the sample that the image ID was associated with.

Reporting for specific materials, systems and methods

We require information from authors about some types of materials, experimental systems and methods used in many studies. Here, indicate whether each material, system or method listed is relevant to your study. If you are not sure if a list item applies to your research, read the appropriate section before selecting a response.

Materials & experimental systems

n/a	Involved in the study	
[ ]	[x]	Antibodies
[x]	[ ]	Eukaryotic cell lines
[x]	[ ]	Palaeontology and archaeology
[ ]	[x]	Animals and other organisms
[ ]	[x]	Human research participants
[x]	[ ]	Clinical data
[x]	[ ]	Dual use research of concern

Methods

n/a	Involved in the study	
[x]	[ ]	ChIP-seq
[ ]	[x]	Flow cytometry
[x]	[ ]	MRI-based neuroimaging

Antibodies

Antibodies used	1. rabbit anti-Ki67: Abcam, Cat # ab15580. Dilution: 1:1000 (IF)<br>2. mouse anti-EpCAM (VU1D9), Cell Signaling Technologies, Cat. # 2929. Dilution: 1:800 (IF)<br>3. goat anti-rabbit Alexa Fluor 488, Invitrogen, Cat. # A32731. Dilution: 1:500 (IF)<br>4. goat anti-mouse Alexa Fluor 568, Invitrogen, Cat. ## A-11004. Dilution: 1:500 (IF)<br>5. FITC-anti-mouse-H-2K/H-2D (clone 34-1-2S), Cat # 114706. Dilution: 1:100 (Flow cytometry)<br>6. APC-anti-human-CD326 (EpCAM), Biolegend, Cat # 324208. Dilution: 1:100 (Flow cytometry)<br>7. Anti-Estrogen Receptor alpha antibody [SP1], Abcam, Cat. # ab16660. Dilution: 1:200 (IHC)<br>8. Mouse Anti-Human Progesterone Receptor (clone PgR 636), Dako, Cat. # M3569. Dilution: 1:200 (IHC)<br>9. HER2 (4B5) Rabbit Monoclonal Primary Antibody, Ventana/Roche, Cat. # 790-4493. Pre-diluted (no dilution in experiment) (IHC)<br>10. Mouse Anti-Human Ki67 Antigen (clone MIB-1), Dako, Cat. # M7240. Dilution: 1:100 (IHC)
Validation	1. https://www.abcam.com/ki67-antibody-ab15580.html<br>2. https://www.cellsignal.com/products/primary-antibodies/epcam-vu1d9-mouse-mab/2929<br>3. https://www.thermofisher.com/antibody/product/Goat-anti-Rabbit-IgG-H-L-Highly-Cross-Adsorbed-Secondary-Antibody-Polyclonal/A32731

4. https://www.thermofisher.com/antibody/product/Goat-anti-Mouse-IgG-H-L-Cross-Adsorbed-Secondary-Antibody-Polyclonal/A-11004
5. https://www.biolegend.com/en-us/products/fitc-anti-mouse-h-2kd-h-2dd-antibody-1886
6. https://www.biolegend.com/en-us/products/apc-anti-human-cd326-epcam-antibody-3758
7. https://www.abcam.com/estrogen-receptor-alpha-antibody-sp1-ab16660.html
8. https://www.agilent.com/en/product/immunohistochemistry/antibodies-controls/primary-antibodies/progesterone-receptor-(concentrate)-76579
9. https://diagnostics.roche.com/global/en/products/tests/ventana-anti-her2-neu-4b5-rabbit-monoclonal-primary-antibody-ce-ivd.html#productInfo
10. https://www.agilent.com/en/product/immunohistochemistry/antibodies-controls/primary-antibodies/ki-67-antigen-(concentrate)-76646

Animals and other organisms

Policy information about studies involving animals; ARRIVE guidelines recommended for reporting animal research

Laboratory animals: NSG mice (NOD.Cg NSG), Females at 4-6wks of age, were used for PDX experiments. Mice were housed in cages containing up to 5 animals on vented racks with 12/12 hour light/dark cycle at 21-22 °C and 35-40% relative humidity.

Wild animals: No wild animals were used in this study.

Field-collected samples: No field-collected samples were used in this study.

Ethics oversight: Ethics oversight was provided by the Research Ethics Board at the Princess Margaret Cancer Centre, University Health Network (UHN, #15-9481).

Note that full information on the approval of the study protocol must also be provided in the manuscript.

Human research participants

Policy information about studies involving human research participants

Population characteristics: All participants were breast cancer patients at the Princess Margret Cancer Center. We included detailed characteristics in supplementary table 2, including diagnosis and treatment history.

Recruitment: Patient tumor tissue was collected with informed patient consent and used according to UHN Research Ethics Board approved protocols (UHN, #06-196 and 15-9481).

Ethics oversight: Ethics oversight was provided by the Research Ethics Board at the Princess Margaret Cancer Centre, University Health Network (UHN, #15-9481 and 06-196).

Note that full information on the approval of the study protocol must also be provided in the manuscript.

Flow Cytometry

Plots

Confirm that:

☒ The axis labels state the marker and fluorochrome used (e.g. CD4-FITC).

☒ The axis scales are clearly visible. Include numbers along axes only for bottom left plot of group (a 'group' is an analysis of identical markers).

☒ All plots are contour plots with outliers or pseudocolor plots.

☒ A numerical value for number of cells or percentage (with statistics) is provided.

Methodology

Sample preparation: Organoids were dissociated to single cells, with TrypLE Express, washed with PBS containing 2% FBS and stained with 1:100 dilution anti-FITC-H2K and 1:100 dilution anti-APC-human EpCAM for 20 minutes, on ice, covered. Samples were washed with PBS containing 2% FBS and resuspended in 500uL PBS with 2% FBS/sample prior to flow cytometry analysis. Unstained controls were included to set gates.

Instrument: BD FACSCanto II Cell Analyzer

Software: FlowJo

Cell population abundance: A minimum of 10,000 gates events were collected. Mouse cell content was measured as the percentage of H2K positive/EpCAM negative cells while human cell content was measured at the percentage of H2K negative/EpCAM positive cells.

Gating strategy: Samples were first gated on FSC/SSC to remove debris. Singlets were then selected using FSC-H and FSC-W. Finally, the percentage of EpCAM and H2K positive and negative cells were determined by setting quadrant gates such that the

unstained control contained no positive events.

☒ Tick this box to confirm that a figure exemplifying the gating strategy is provided in the Supplementary Information.
